# Revealing Spatial Molecular Heterogeneity of High-Density Biofunctionalized Surfaces Using DNA-PAINT

**DOI:** 10.1021/acsami.4c10310

**Published:** 2024-10-21

**Authors:** Wei Shan Tan, Arthur M. de Jong, Menno W. J. Prins

**Affiliations:** † Department of Biomedical Engineering, Eindhoven University of Technology, Eindhoven 5612 AZ, The Netherlands; ‡ Institute for Complex Molecular Systems (ICMS), Eindhoven University of Technology, Eindhoven 5612 AZ, The Netherlands; § Department of Applied Physics, Eindhoven University of Technology, Eindhoven 5612 AZ, The Netherlands; ∥ Helia Biomonitoring, Eindhoven 5612 AR, The Netherlands

**Keywords:** high-density biofunctionalization, biomolecule quantification, DNA-PAINT, super-resolution imaging, single-molecule

## Abstract

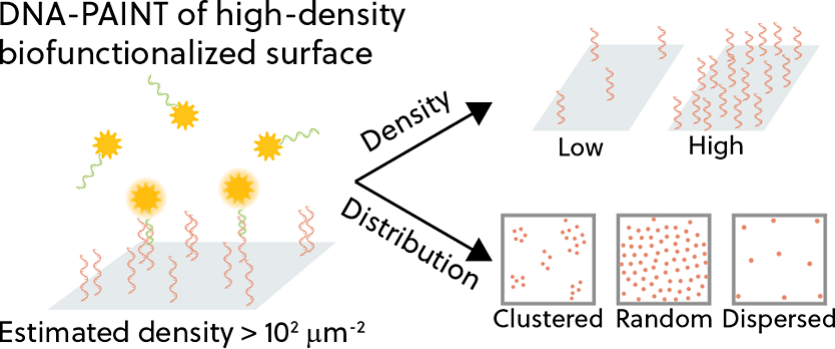

The quantification and control of molecular densities and distributions on biofunctionalized surfaces are key for enabling reproducible functions in biosciences. Here, we describe an analysis methodology for quantifying the density and spatial distribution of high-density biofunctionalized surfaces, with densities in the order of 10^2^–10^5^ biomolecules per μm^2^ area, in a short measurement time. The methodology is based on single-molecule DNA-PAINT imaging combined with simulation models that compensate for lifetime and spatial undersampling effects, resulting in three distinct molecule counting methods and a statistical test for spatial distribution. The analysis methodology is exemplified for a surface with ssDNA affinity binder molecules coupled to a PLL-*g*-PEG antifouling coating. The results provide insights into the biofunctionalization efficiency, yield, and homogeneity. Furthermore, the data reveal that heterogeneity is inherent to the biofunctionalization process and shed light on the underlying molecular mechanisms. We envision that DNA-PAINT imaging with the developed analysis framework will become a versatile tool to study spatial heterogeneity of densely biofunctionalized surfaces for a wide range of applications.

## 1 Introduction

Surface modifications to enable biological and biochemical functionalities, termed biofunctionalization, are a central theme in many fields of study, including regenerative medicine, targeted drug delivery, and bioanalytical applications. Here, surfaces are provided with affinity molecules in order to effectuate specific interactions with molecules, cells, and tissues.^1−3^ The densities of surface-bound affinity molecules are typically high, in the range of 10^2^–10^5^ μm^–2^, corresponding to mean intermolecular distances between 50 and 2 nm. For functionality and reproducibility, it is important to know and control the spatial distribution of the molecules on the surface. However, it is difficult to quantitatively evaluate the spatial distributions due to the very high densities of the affinity molecules on the surface. Well-known characterization techniques such as quartz crystal microbalance^4^ and surface plasmon resonance^5^ give average densities of molecules, but not their spatial heterogeneity. Widefield fluorescence imaging techniques allow qualitative comparisons but are not suited for quantifying densities and heterogeneity.

A special class of fluorescence imaging techniques, known as single-molecule localization microscopy (SMLM), presents new avenues toward the quantification of biomolecules on surfaces with single-molecule resolution. SMLM techniques separate fluorescent emissions of single fluorophores or fluorescent dyes in time, causing digital events with on–off behavior. The temporal separation avoids spatial overlaps between the point spread functions (PSFs) of individual fluorophores so that spatial coordinates of each fluorophore can be determined with high precision.^6^ By sequentially imaging the fluorophores, a super-resolution image is obtained and the spatial location of individual biomolecules can be extracted.

DNA point accumulation in nanoscale topography (DNA-PAINT) is particularly attractive because of its relative simplicity in instrumental implementations and the use of DNA probes with well-controlled event kinetics.^7,8^ In this case, the temporal separation of fluorophores is achieved by the transient binding of dye-labeled single-stranded DNA (ssDNA), known as the imager strands, to complementary ssDNA on the surface, known as docking strands. To gauge the number of biomolecules in a resolution-limited area, quantitative DNA-PAINT (qPAINT) can be used to count the number of biomolecules by analyzing the DNA hybridization kinetics.^9,10^ In recent years, works based on DNA-PAINT imaging have demonstrated the quantification of molecular densities up to a few hundreds of molecules per μm^2^,^10^ and have studied the molecular distribution of molecules that are spaced more closely than the standard DNA-PAINT resolution.^11^ However, with these methodologies, the analysis of high surface densities would require very long measurement times (days) and give limited statistics.

In this article, we describe the development and application of a data analysis framework based on standard DNA-PAINT imaging to obtain insights into the molecular density and distribution of densely biofunctionalized surfaces. The surfaces are analyzed and quantified from single-molecule imaging data collected in a short time (∼1 h), by using simulation models that are able to compensate for lifetime and spatial undersampling effects. The analysis is demonstrated for a ssDNA-functionalized surface based on a low-fouling poly(l-lysine)-grafted-poly(ethylene glycol) (PLL-*g*-PEG) coating.^12,13^ The DNA-PAINT analysis of this molecular system reveals unexpected spatial molecular heterogeneity of ssDNA affinity binders that depends strongly on the biofunctionalization conditions.

## 2 Results and Discussion

### 2.1 DNA-PAINT: Experimental and Analysis Consideration

**Figure 1:**
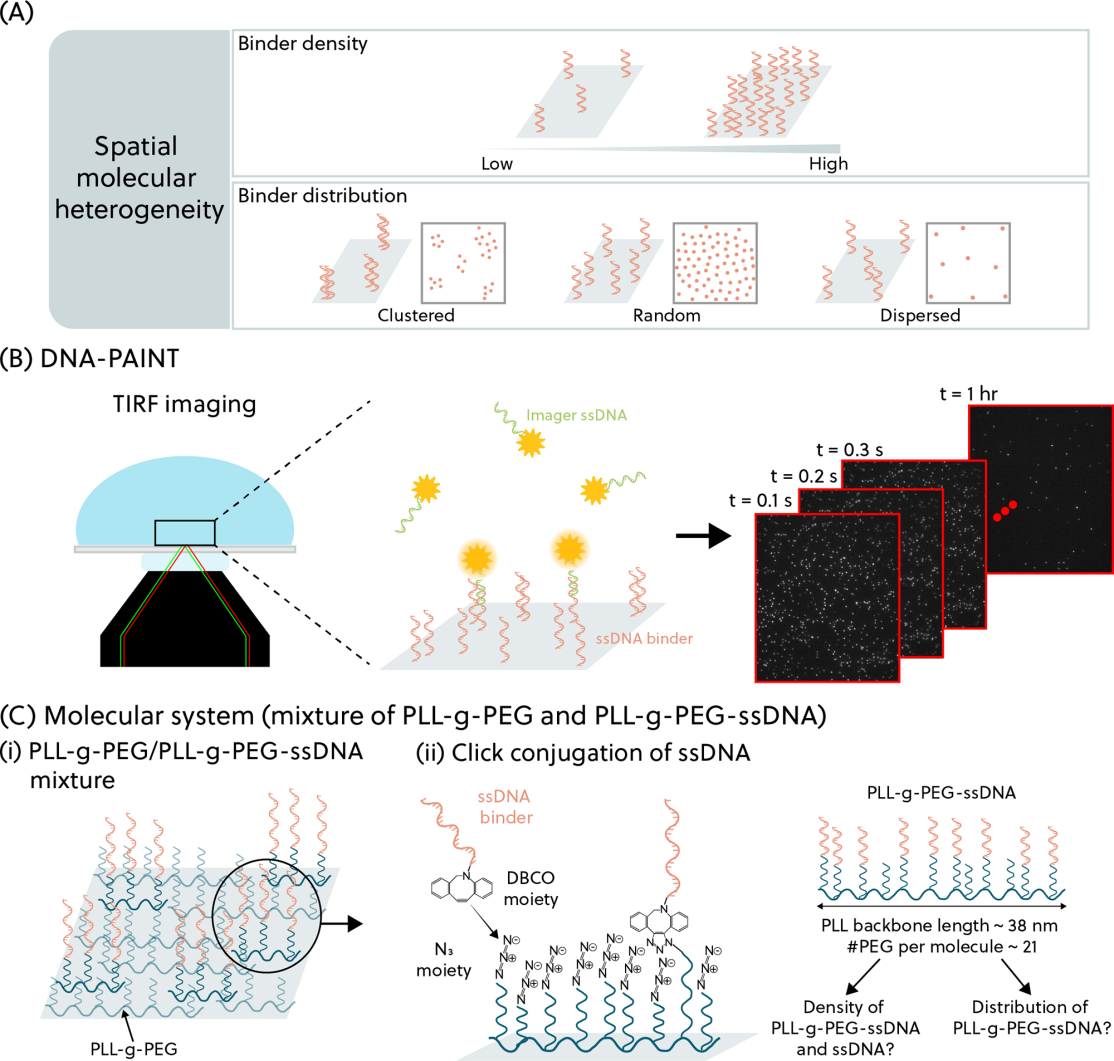
(A) Spatial molecular heterogeneity refers to heterogeneity that results from differences in binder density (areas with varying binder densities) and/or nonrandom binder distribution. (B) DNA-PAINT is performed under total internal reflection conditions where a thin layer at the substrate surface is optically excited. ATTO647N labeled imager strands are designed to have a 10-base pair complementarity to the ssDNA binders. Transient binding interactions result in fluorescence events that are recorded at a frame rate of 10 Hz for 1 h. (C) (i) The molecular system employed in this study is a mixture of low-fouling polymer PLL-*g*-PEG and ssDNA-functionalized PLL-*g*-PEG. (ii) DBCO-functionalized ssDNA binders are click-conjugated to the azide-functionalized PLL-*g*-PEG molecules that have been coated on the surface. On average, the PLL backbone contour length of the PLL-*g*-PEG-ssDNA molecule is estimated to be 38 nm and the molecule has approximately 21 PEG side chains, all of which are functionalized with azide moieties and can be further functionalized with ssDNA binders.

DNA-PAINT is an SMLM technique that makes use of sequence-dependent DNA interactions to achieve super-resolution imaging. Figure 1B illustrates the working principle of DNA-PAINT. To probe the surface-bound ssDNA binders, the imager strand is designed to have a complementarity of 10 nucleotides with the binders. The hybridization time (the time duration in which a fluorescent signal is observed) is then estimated to be in the order of a few seconds.^7^ To maintain a high signal-to-noise ratio for such experiments, DNA-PAINT is performed using a total internal reflection fluorescence (TIRF) setup. Here, only dye-labeled imager strands close to and on the surface are excited by the evanescent field of the reflected light. This reduces the background, since only fluorescent signal is detected from imager molecules close to the substrate. The temporally separated binding events are then captured frame-by-frame with a frame rate of 10 Hz and postprocessed to obtain spatial, intensity, and other information on each detected fluorophore in each frame.

In this work, DNA-PAINT imaging is used to study the spatial molecular heterogeneity of ssDNA affinity binders functionalized on a PLL-*g*-PEG-coated glass substrate. The model system at the molecular scale is a mixture of PLL-*g*-PEG molecules and ssDNA-functionalized PLL-*g*-PEG molecules, as shown in Figure 1C. The glass substrate is first coated with a mixture of PLL-*g*-PEG and azide-functionalized PLL-*g*-PEG via electrostatic physisorption, then the dibenzocyclooctyne-functionalized ssDNAs (DBCO-ssDNAs) are conjugated to the azide-functionalized PLL-*g*-PEG via Strain Promoted Azide Alkyne Cycloaddition (SPAAC) click chemistry. The model system is chosen because PEG-based systems are widely employed in the fields of regenerative medicine, targeted drug delivery and bioanalytical systems,^14−16^ and are thus relevant for these applications. Furthermore, this system presents a few interesting complexities:

- The azide-functionalized PLL-*g*-PEG molecule (to which the ssDNA molecules are conjugated) has a PLL backbone chain contour length of approximately 38 nm. The polymer chains can coil as they attach to and rearrange on the glass surface, resulting in smaller effective chain lengths. This means that imaging individual PLL-*g*-PEG-ssDNA molecules is near or below the resolution limit of DNA-PAINT.
- It is not known how many ssDNA binders are coupled to individual azide-functionalized PLL-*g*-PEG molecules. We estimate about 21 azide-functionalized PEG side chains in one PLL-*g*-PEG molecule (see Supplementary Section 1). In other words, depending on the biofunctionalization conditions, there may be between 0 and 21 ssDNA binders per azide-functionalized PLL-*g*-PEG molecule, i.e., up to 21 ssDNA binders in a resolution-limited area. Thus, both the molecular densities of whole chains of PLL-*g*-PEG-ssDNA and of individual ssDNA binders on the substrate should be investigated.
- In case of biofunctionalization conditions that yield 2 or more ssDNA binders per PLL-*g*-PEG molecule, the molecular distribution of the ssDNA binders on a given surface area is inherently clustered. Rather than the molecular distribution of individual ssDNA binders, the molecular distribution of PLL-*g*-PEG-ssDNA molecules on the surface becomes a more interesting property to be investigated, since it is the nanoscale organization of the PLL-*g*-PEG-ssDNA molecules that determines the overall spatial arrangement of the ssDNA binders on a larger scale.

These molecular complexities demand a single-molecule characterization technique that is capable of analyzing densely functionalized samples.

**Figure 2:**
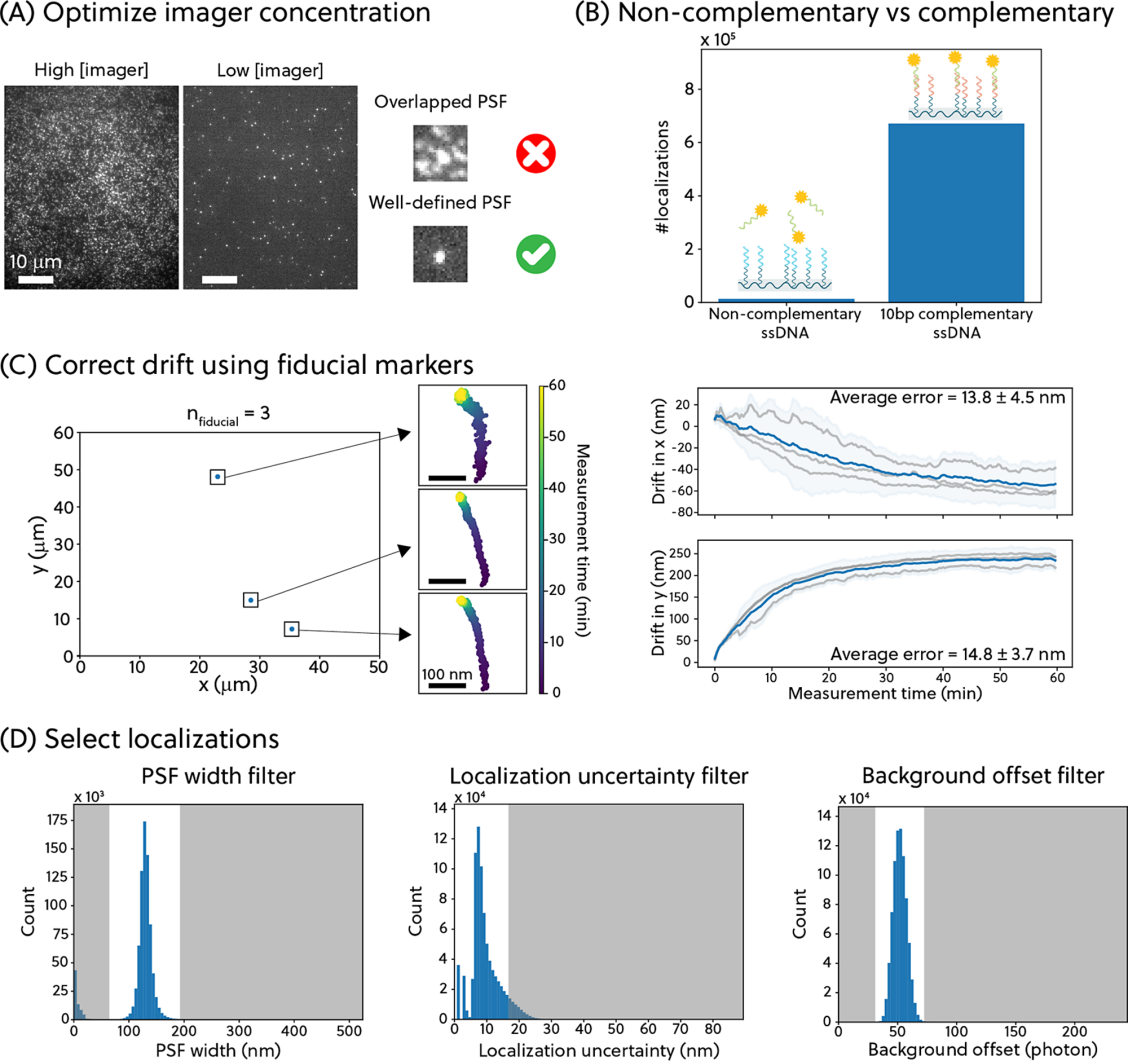
(A) Imager concentration is optimized to avoid spatial overlap of PSFs. Well-defined PSFs give confidence to the quality of localizations obtained in downstream analysis. (B) Experimental control using noncomplementary ssDNA (no pairing to the imager strands) functionalized on a PLL-*g*-PEG-coated surface shows a low number of localizations as compared to that with complementary ssDNA binders. (C) Measurement drift is corrected using fluorescent particles as fiducial markers. Drifts in the *x*- and *y*-direction for each fiducial marker (gray lines) are averaged and used to correct drift for all localizations (the average drift is shown as the blue line). The average errors in drift for the entire measurement duration (±standard deviation) are quantified as (13.8 ± 4.5) and (14.8 ± 3.7) nm, for *x*- and *y*-direction, respectively. (D) To remove false localizations, three filters based on PSF width, localization uncertainty, and background offset have been employed.

There are a few practicalities to consider when performing DNA-PAINT experiments for surfaces with high binder densities as detailed in Figure 2. The first and most important to consider is the imager concentration. To image low-density surfaces using DNA-PAINT, the imager strands are typically used at concentrations of 0.1–1 nM. In this scenario, the imager strands are in excess as compared to the docking strands, so the spatial locations of the docking strands are determined by recurring binding events on the same docking sites. If one were to perform DNA-PAINT imaging under the same condition for samples with high binder densities, the raw images would be saturated with fluorescent emissions, and their PSFs would be spatially overlapping, see Figure 2A. Although it is possible to use multi-emitter fitting analysis to localize overlapping PSFs, this analysis involves a large computational cost and localization uncertainties.^17,18^ Therefore, we opted to set the imager concentration lower (25 pM) to avoid saturation of the raw fluorescence images. To ensure sufficient precision of the spatial localizations, the microscopy images were acquired over a period of 1 h.

Next, it is crucial to establish experimental controls to quantify background signals, in order to verify the specificity of the interaction between the imager strands and the ssDNA binders. In the case of imaging DNA origami or biofunctionalized particles, background signals can be easily quantified by distinguishing between localizations that relate to the shape of the underlying structure and those not related to the shape.^19,20^ However, on a flat surface, no shape can help to distinguish whether the observed binding events are specific or not. In Figure 2B, the negative control (PLL-*g*-PEG-coated surfaces functionalized with ssDNA molecules not complementary to the imager strands) shows a significantly lower number of localizations as compared to that functionalized with the ssDNA binders, giving confidence that the localizations observed on the surface with complementary strands are caused by specific interactions. The localizations are obtained from ThunderSTORM,^32^ an open-source ImageJ plugin that analyzes single-molecule fluorescence data. Since the percentage of nonspecific localizations to specific localizations is approximately 2%, all localizations in the experimental data are analyzed and are treated as specific interactions.

To accurately determine the spatial locations of the surface-coupled binders, the effect of drift (resulting from temperature changes or mechanical vibrations) must be accounted for. Thus, fiducial markers were added as reference points to perform drift correction. There are typically a few fiducial markers in the field of view that are utilized for drift correction, see Figure 2C. The relative *xy*-position of every frame with respect to a reference frame is computed for each marker. The drifts of all fiducial markers are then averaged to give the average *x*- and *y*-drift, and the average drift is deducted from all localizations.

Furthermore, the localizations were filtered according to the width of their PSF, the localization uncertainty, and the background offset, see Figure 2D. PSF width provides information on the shape of the PSF of the fluorophore to exclude localizations with unusually large or small PSF widths. Localization uncertainty, also known as localization precision, is the uncertainty of a lateral position estimate of the detected localization. The criterion excludes localizations with uncertainties larger than twice the median uncertainty value. Lastly, background offset is the difference in intensity between the background of the localization and the camera baseline and is used to exclude falsely identified localizations.

### 2.2 Quantifying Molecular Density of PLL-*g*-PEG-ssDNA and ssDNA

**Figure 3:**
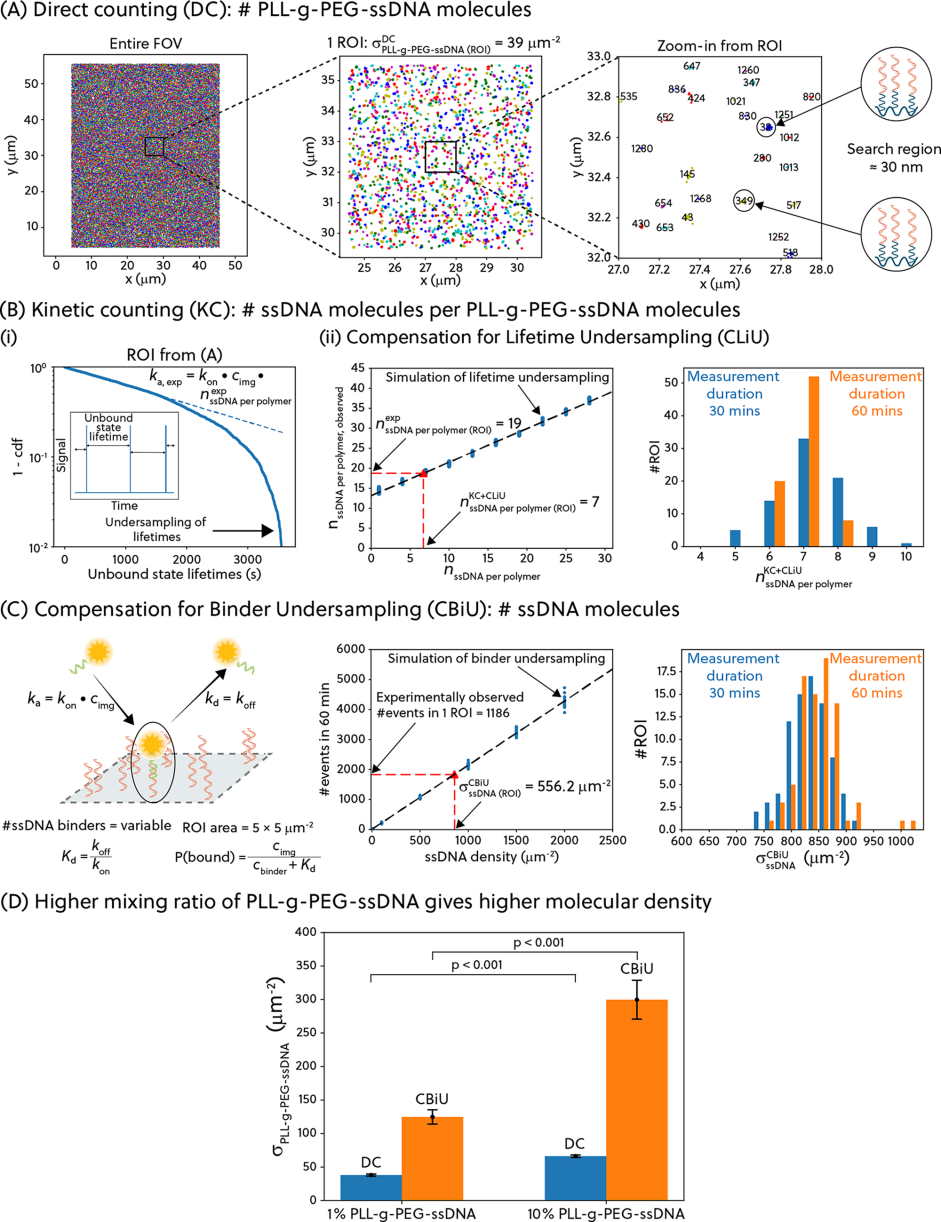
(A) Direct counting approach based on the mean-shift clustering algorithm to quantify the number of PLL-*g*-PEG-ssDNA molecules in a FOV. (B) Kinetic counting approach, compensated for lifetime undersampling, to estimate the average number of origami-equivalent ssDNA binders per PLL-*g*-PEG-ssDNA molecule. Cumulative distribution function is denoted as cdf. (C) Compensation for binder undersampling (CBiU) analysis to estimate the total number of ssDNA binders in an ROI. (D) By increasing the mixing ratio of PLL-*g*-PEG-ssDNA from 1% to 10%, a significant increase (*p* ≤ 0.1%) in the density of PLL-*g*-PEG-ssDNA molecules is observed for all quantification methods (mean ± std).

Three analysis methods were established to quantify the density of the PLL-*g*-PEG-ssDNA molecules and ssDNA binders in a given region of interest (ROI) as shown in Figure 3. Every biofunctionalized sample is imaged in a field of view (FOV) with an area of approximately 50 × 60 μm^2^. The FOV is then divided into 120 ROIs of 5 × 5 μm^2^. The ROIs at the edge of the FOV are discarded to avoid edge effects, so the remaining 80 ROIs are used for further analysis.

Looking further into the localizations within each ROI in Figure 3A, the binding events (which individually may last for a few seconds) are observed as clouds of localizations in a time-aggregated localization plot. This cumulative plot ignores the precise times at which the localizations occurred. The localization clouds indicate where the imager strands have interacted with the ssDNA-functionalized PLL-*g*-PEG molecules. To identify the localization clouds and estimate their center position, a direct counting (DC) method based on the mean-shift clustering algorithm is applied to the data.^21−23^ This algorithm uses a bandwidth parameter that relates to the expected size of the localization cloud. Using an inappropriate bandwidth parameter would result in falsely identified localization clouds (see Supplementary Section 3.1). For a biofunctionalized, flat surface, the size of the localization cloud is given by the DNA-PAINT localization uncertainty, thus the bandwidth was set to twice the maximum value of the localization uncertainty (≈30 nm). The obtained center positions of the localization clouds are interpreted as the estimated spatial locations of each PLL-*g*-PEG-ssDNA molecule and are used to compute the direct counted (DC) density of whole-polymer PLL-*g*-PEG-ssDNA molecules σ_PLL-g-PEG-ssDNA_^DC^. The assumption that each localization cloud represents a PLL-*g*-PEG-ssDNA molecule is supported by the fact that the expected distance between the PLL-*g*-PEG-ssDNA molecules is larger than the DNA-PAINT resolution limit (see Supplementary Section 1) for the mixing ratio of PLL-*g*-PEG-ssDNA molecules employed in this study.

Further zooming in on each localization cloud, the localizations within the localization cloud contain temporal information, i.e., at which frames individual localizations were found. Similar to a qPAINT analysis, temporal information can be used to determine the number of ssDNA binders functionalized on a single PLL-*g*-PEG-ssDNA molecule. This approach is termed the kinetic counting (KC) approach. The experimental association rate, *k*_a_^exp^, can be computed by analyzing the signal-time traces, extracting the unbound state lifetimes (the time duration in which no fluorescent signal is observed) for each localization cloud, and pooling the unbound state lifetimes from all the localization clouds in one ROI (see Figure 3B(i)). The association rate is related to the experimental average number of ssDNA binders per PLL-*g*-PEG-ssDNA molecule in one ROI, *n*_ssDNA per polymer_^exp^, according to eq 1:
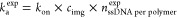
1where *k*_on_ denotes the association rate between the imager strand and the ssDNA binders, and *c*_img_ the imager concentration. In many qPAINT studies, *k*_on_ has been quantified to be in the order of 10^6^ M^–1^s^–1^ using surfaces with well-defined single ssDNA docking strands (typically on a DNA origami), thus we assume a *k*_on_ reference value of 10^6^ M^–1^s^–1^ in this study. Therefore, the number of ssDNA determined in this study should be interpreted as the average number of origami-equivalent ssDNA binders per PLL-*g*-PEG-ssDNA molecule. The impact of *k*_on_ on the quantification of the average number of ssDNA binders per PLL-*g*-PEG-ssDNA molecule is detailed in Supplementary Section 3.2.

Figure 3B(i) illustrates the undersampling issue that arises from imaging with low imager concentration in a limited time. Due to the experimental requirements for high-density samples, most of the localization clouds have only a few binding events in the entire measurement duration. This causes a bias toward short unbound state lifetimes as we can only observe an unbound state lifetime that is shorter than the measurement duration (1 h). To compensate for the undersampling of lifetimes, Monte Carlo simulations were performed to investigate the effect of undersampling on the quantification (the simulation is detailed in Supplementary Section 3.2). This procedure is termed the Compensation for Lifetime Undersampling (CLiU). The relationship obtained is then used to calculate the compensated average number of origami-equivalent ssDNA binders per PLL-*g*-PEG-ssDNA molecule, *n*_ssDNA per polymer_^KC+CLiU^, as expressed in eq 2:
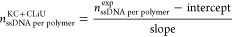
2where slope and intercept are the linear fit parameters with values of 0.8 and 13.0 respectively for the given experimental conditions. Combined with the PLL-*g*-PEG-ssDNA density obtained from the DC analysis, the density of ssDNA binders in an ROI, σ_ssDNA_^KC+CLiU^, can be determined by the expression in eq 3:
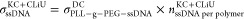
3To verify that the derived number is independent of the measurement duration, we performed the CLiU analysis on datasets with different measurement durations. On the full 60 min dataset and a 30 min subset of the dataset, most ROIs were quantified to have 7 origami-equivalent ssDNA binders per PLL-*g*-PEG-ssDNA molecule. The slightly broader distribution of the quantified *n*_ssDNA per polymer_^KC+CLiU^ in the 30 min dataset arises from the reduced statistics in the shorter measurement.

Other than the undersampling of lifetimes, we also observed an undersampling of binder molecules as a consequence of the imaging conditions. The number of localization clouds was found to increase with increasing measurement time, indicating that more ssDNA binders are still being sampled in the 1-h measurement, see Supplementary Section 3.3. A way to mediate this could be to perform measurements even longer than 1 h. However, it is impractical to perform hours-long DNA-PAINT experiments for a single sample. Hence, we explored a Monte Carlo simulation approach, termed the Compensation for Binder Undersampling (CBiU) analysis, to estimate the total number of binders in an ROI, as shown in Figure 3C. Briefly, a time trace is simulated for each ssDNA binder for varying ssDNA density. The hybridization parameters related to association *k*_a_ and dissociation *k*_d_ are given by eq 4:
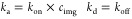
4where the dissociation rate *k*_off_ is experimentally determined to be approximately 1 *s*^–1^, see Supplementary Figure S3. Each ssDNA binder has a probability of being visited and bound to an imager strand, according to eq 5:
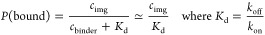
5where *c*_binder_ and *K*_d_ denote the effective volumetric binder concentration and origami-equivalent equilibrium dissociation constant, respectively. This equation is valid with the conditions that the ssDNA binders are in excess as compared to the imager strands and that the equilibrium dissociation constant is much larger than the binder and imager concentration (*c*_binder_ ≫ *c*_img_, *K*_d_ ≫ *c*_binder_, *K*_d_ ≫ *c*_img_).^24^ For the molecular system considered here, *c*_binder_ and *K*_d_ are estimated to be in the nM range and the μM range respectively, while *c*_img_ is set at 25 pM experimentally, see Supplementary Section 1.

By performing CBiU analysis, we investigated the relationship between the density of ssDNA binders and the total number of binding events observed within the simulated duration (1 h). We then leveraged this relationship to determine the ssDNA density in an ROI, σ_ssDNA_^CBiU^, based on the total number of binding events experimentally observed in the ROI, as seen in Figure 3C (middle). The CBiU-estimated ssDNA density can also be combined with the KC approach to compute the number of PLL-*g*-PEG-ssDNA in the ROI, i.e. as expressed in eq 6:
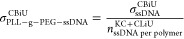
6As in the CLiU analysis, we showed that similar quantification is obtained from the CBiU analysis for the same dataset independent of the measurement duration, as seen in Figure 3C(rightmost panel).

Figure 3D shows the capability of the analysis to quantify the density of PLL-*g*-PEG-ssDNA molecules. By increasing the mixing ratio of azide-functionalized PLL-*g*-PEG (hence the ratio of PLL-*g*-PEG-ssDNA molecules to total PLL-*g*-PEG molecules), we observed a significantly higher density of PLL-*g*-PEG-ssDNA molecules. However, it is intriguing to see that a 10-fold increase in the mixing ratio results in a roughly 2-fold increase in PLL-*g*-PEG-ssDNA density. This suggests that varying the mixing ratio does not necessarily result in a proportional increase in binder density, highlighting the necessity of performing biomolecule quantification when optimizing biofunctionalization processes. We hypothesize that the observation is caused by the molecular heterogeneity of the polymer molecules (varying PLL backbone length, grafting ratio, etc*.*, see Supplementary Section 1), resulting in a more favorable attachment of the nonfunctionalized PLL-*g*-PEG molecules. The hypothesis is not further investigated in this article and will be studied in later works. Furthermore, the comparison between σ_PLL-g-PEG-ssDNA_^DC^ and σ_PLL-g-PEG-ssDNA_^CBiU^ provides insights into the extent of undersampling in the DC quantification. For the samples containing 1% and 10% mixing ratio of PLL-*g*-PEG-ssDNA, (31 ± 3)% and (22 ± 2)% of PLL-*g*-PEG-ssDNA molecules were probed and quantified via the DC analysis, respectively.

In theory, the analysis methods can be applied to samples of even higher molecular densities provided that the distance between the molecules, to which the ssDNA binder molecules were functionalized, is within the resolution limit of DNA-PAINT. If the distance is smaller than the resolution limit, the densities, quantified using the KC approach and compensated using the CLiU and CBiU analysis, could still provide quantitative information regarding the molecular density of the binder molecules, but the interpretation of the DC density must be reconsidered. Overall, for the model system in this work, we demonstrated that DNA-PAINT can be used to quantify the high molecular density of PLL-*g*-PEG-ssDNA and ssDNA binder molecules with a relatively short measurement time (1 h) by compensating for the undersampling effects.

### 2.3 Quantifying Molecular Distribution of PLL-*g*-PEG-ssDNA

**Figure 4:**
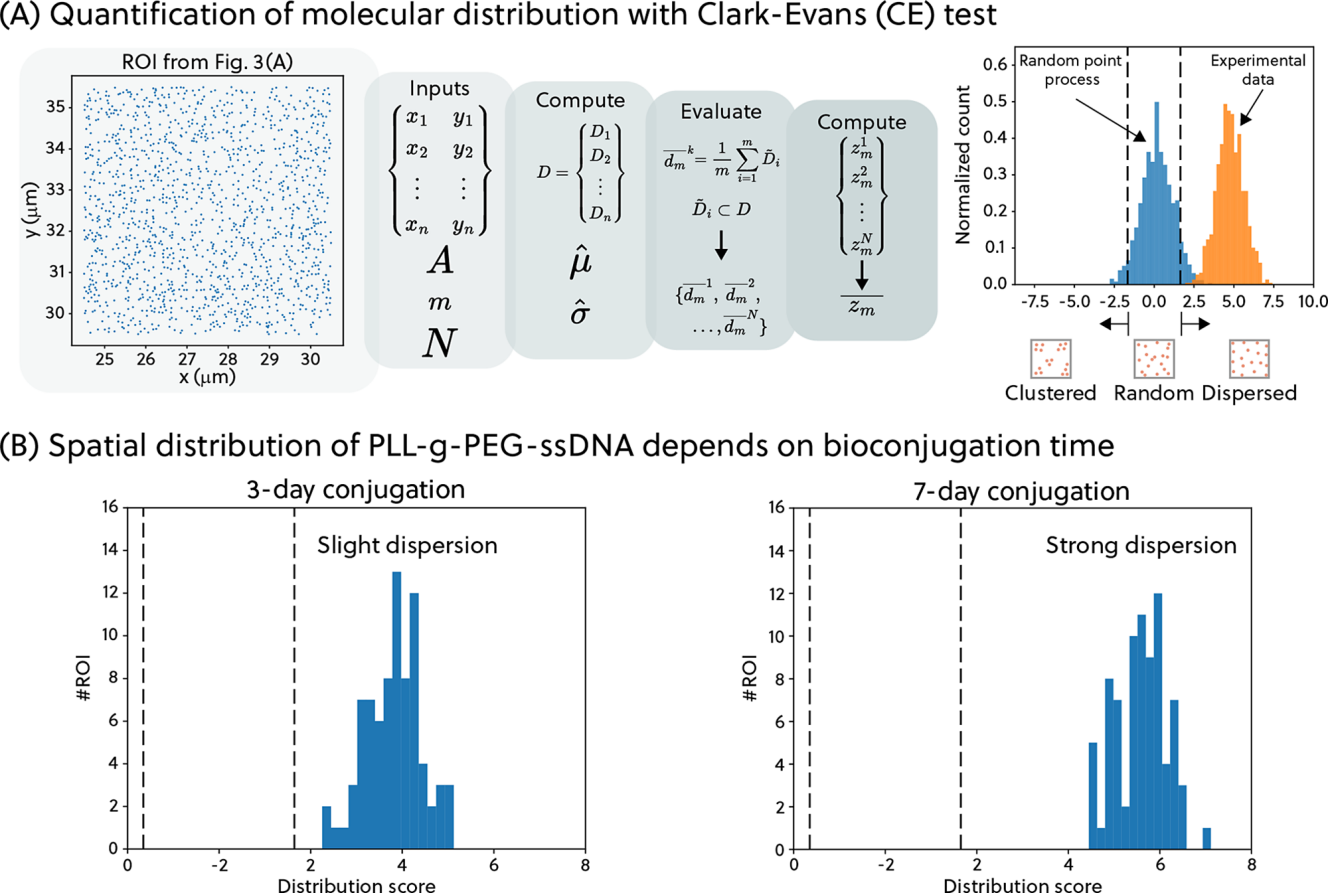
(A) Step-by-step analysis to quantify molecular distribution using the Clark–Evans test. By comparing the experimentally observed distribution score to that of the random point process (CSR point pattern), the distribution of the PLL-*g*-PEG-ssDNA molecules in an ROI can be quantified. (B) Increasing the ssDNA conjugation time results in an increase in the dispersion of the PLL-*g*-PEG-ssDNA molecules.

To quantify the spatial distribution of binders on surfaces, a reference distribution based on the complete spatial randomness (CSR) hypothesis is used. CSR describes a completely random point pattern, synonymous with a homogeneous Poisson point process that only depends on the density of points. Statistical tests have been developed to study whether observed point patterns deviate from the CSR hypothesis. Here, we use a nearest-neighbor distance-based test proposed by Clark and Evans to analyze and quantify the spatial distribution of PLL-*g*-PEG-ssDNA molecules.^25^ Nearest-neighbor distance (NND) is the distance between a point and its closest neighbor. To construct the test, the DC-estimated spatial locations of PLL-*g*-PEG-ssDNA molecules, the area *A* in which the molecules are present, the number of sampled NNDs *m* taken to ensure the independence of NND, and the number of sampling *N* are taken as inputs. The rationale behind the analysis is explained in the Supplementary Section 3.4. In brief, *N* subsets of NNDs of the estimated spatial locations are computed and averaged to give the sample mean NND , where *k* ∈ [1, *N*]. As shown in eq 7, these values are then standardized to give,
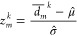
7where μ̂ and σ̂ denote the mean and standard error of NND of a CSR point pattern. μ̂ and σ̂ are computed by using eq 8,
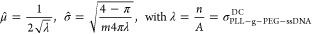
8where *n* is the number of estimated molecules. By averaging the obtained *z*_*m*_^*k*^ values, the standardized mean NND , denoted as the distribution score in this work, is used to evaluate whether the observed molecular distribution is plausible under CSR, i.e., whether the observed distribution is indeed random. To indicate whether the molecules are more clustered or more dispersed than random, the  needs to be tested against the distribution score of a CSR point pattern at a certain significance level α. In this work, a 5% significance is chosen as standard, hence  is checked against *z*_0.05_ = 1.65. As shown in Figure 4A, the interpretation of  is summarized as in eq 9:
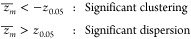
9

To study the effect of undersampling on the quantification of the molecular distribution, both random and nonrandom point patterns with varying degrees of clustering and dispersity have been generated, see Supplementary Section 3.4. In general, the undersampling of molecules reduces how significantly the true molecular distribution deviates from the CSR. Therefore, the distribution score quantified in this work is interpreted only as an indication of the true molecular distribution. Interestingly, it appears that the impact of undersampling on the quantification of the molecular distribution is less pronounced for high molecular density than for low molecular density. Since we quantified σ_PLL-g-PEG-ssDNA_^CBiU^ in the range of 10^2^ μm^–2^ (σ_ssDNA_^CBiU^ up to 10^3^ μm^–2^), we do not expect the undersampling to impact the quantification of the distribution source.

By performing this analysis on samples of varying ssDNA bioconjugation time, we observed an increasing dispersity of the PLL-*g*-PEG-ssDNA molecules as the bioconjugation time increases, see Figure 4B. It is not a priori clear what could cause the increasing dispersion over time. We hypothesize that this observed increase in dispersity could be caused by either the steric repulsion between the PLL-*g*-PEG-ssDNA molecules that are getting increasingly saturated with ssDNA binders over time, or by the inherent molecular distribution of the azide-functionalized PLL-*g*-PEG molecules, or by a combination of both. These hypotheses are discussed in Supplementary Section 5. The interplay of forces that could result in the observed molecular distributions is not part of this article and will be studied in later works.

### 2.4 Biofunctionalization-Induced Spatial Molecular Heterogeneity

**Figure 5:**
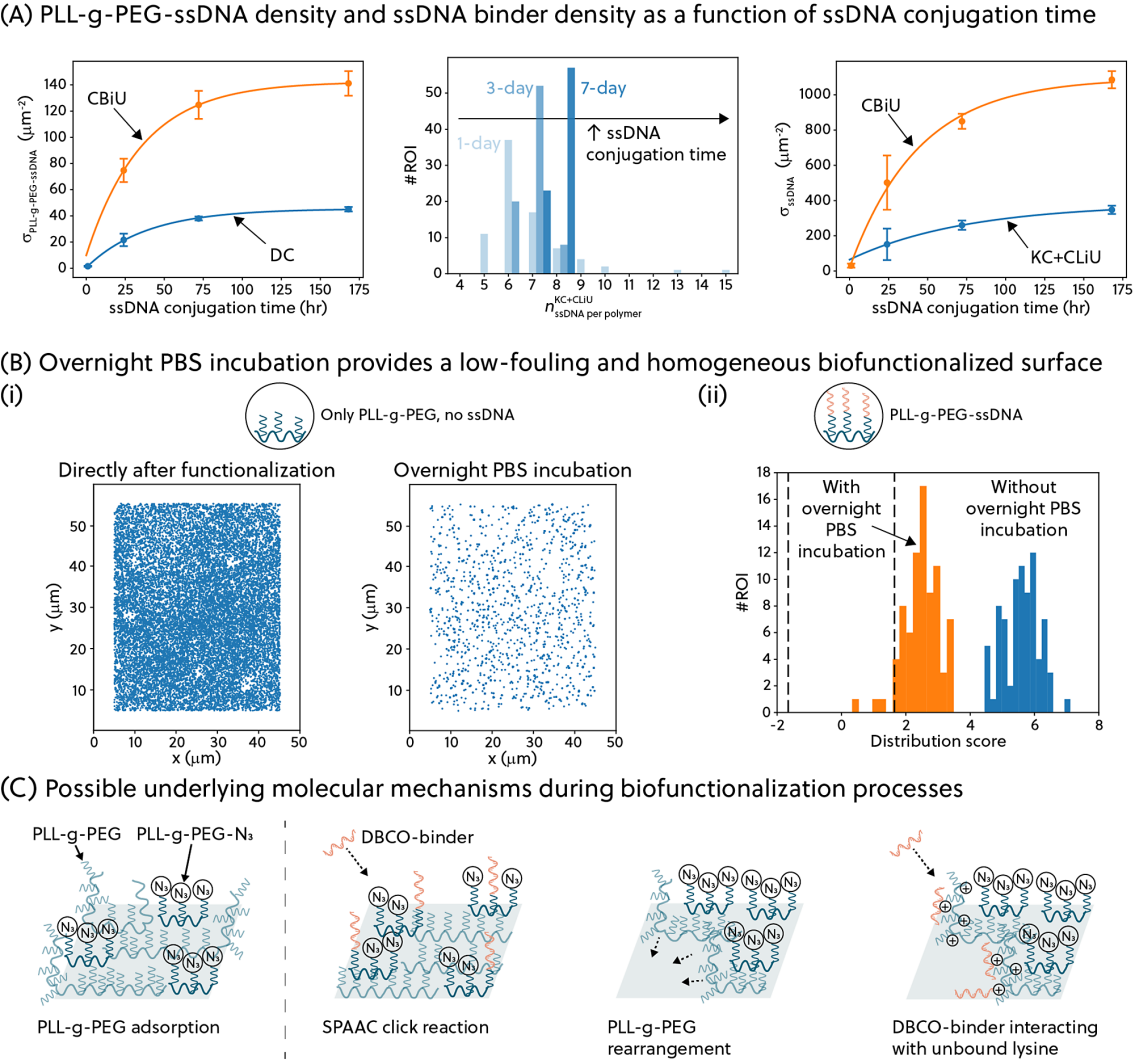
(A) Increasing the ssDNA conjugation time increases the PLL-*g*-PEG-ssDNA density, the average number of ssDNA binders per PLL-*g*-PEG molecule, and the total ssDNA density. Densities are fitted with a single-exponential equation, σ = *a* – *be*^–*x*/τ^ where *a* and *b* are fit parameters related to the equilibrium yield, and τ is related to the conjugation time scale. (B) Overnight PBS incubation, prior to ssDNA conjugation, prepares a better low-fouling surface (i – fewer nonspecific interactions) and a more homogeneous biofunctionalized surface (ii – lower distribution score indicating lower dispersity). (C) Biofunctionalization conditions affect the spatial heterogeneity of surface-bound binder molecules. After PLL-*g*-PEG adsorption, several molecular mechanisms may occur: (1) SPAAC click reaction, (2) PLL-*g*-PEG rearrangement, and (3) DBCO-functionalized binder molecules interacting with unbound lysine molecules. Mechanisms 2 and 3 are hypothesized to be reduced via the overnight PBS incubation step.

The technique and analysis outlined in the previous sections can be used to study whether spatial molecular heterogeneity can arise from varying biofunctionalization conditions. In Figure 5A, we studied the effect of ssDNA conjugation time on the PLL-*g*-PEG-ssDNA density, average number of origami-equivalent ssDNA binders per PLL-*g*-PEG-ssDNA molecule, and the total ssDNA density based on a 1% mixing ratio of azide-functionalized PLL-*g*-PEG. The occurrence of the SPAAC reaction is supported by the observation of progressively reduced nonspecific interactions on the control surfaces, where noncomplementary ssDNA molecules are increasingly conjugated on the surface, as shown in Supplementary Figure S13. Overall, we observe a single-exponential increase in the PLL-*g*-PEG-ssDNA and ssDNA binder densities as a function of bioconjugation time. For all three analysis methods, the characteristic conjugation time τ, obtained from the single-exponential fit parameter, is evaluated to be (48 ± 18) h. The click reaction rate characterized in this work is in agreement with the relatively low second-order reaction rate constants quantified in literature (1–60 M^–1^ s^–1^).^26^

Similar to the previous observation, *n*_ssDNA per polymer_^KC+CLiU^ increases with increasing ssDNA conjugation time. *n*_ssDNA per polymer_^KC+CLiU^ is quantified using the KC and CLiU approach as detailed in the previous section. At short conjugation time (1 day), we observed a larger variability of the quantity in all ROIs, presumably arising from the stochastic conjugation process via SPAAC click chemistry. As conjugation time increases, the variability between ROIs reduces, indicating that the azide-functionalized PLL-*g*-PEG molecules became more saturated with the ssDNA binder molecules over time. After 7 days of click reaction, most ROIs show an average of 8 origami-equivalent ssDNA binders per PLL-*g*-PEG molecule. In theory, there are 21 azide-functionalized PEG molecules available to react with the DBCO-functionalized ssDNA binders. By considering the geometry of the binder molecules, we estimated that approximately 10 binder molecules can physically fit onto one azide-functionalized PLL-*g*-PEG molecule (Supplementary Section 1). We postulate that the discrepancy between the geometrical estimation and the experimental values can stem from several aspects. First, the *k*_on_ reference value is chosen from literature and may not reflect the true molecular picture since it is likely that the accessibility of the docking strands is hindered in a high-density sample, which may reduce the molecular association rate *k*_on_.^27^ In the geometrical estimation, the PLL-*g*-PEG molecules are assumed to be well extended on the surface, but this may not necessarily be the case.

On top of that, our experiments revealed the dynamic rearrangement of the low-fouling PLL-*g*-PEG coatings as shown in Figure 5B. By considering substrates functionalized with PLL-*g*-PEG molecules (no azide-functionalized PLL-*g*-PEG and ssDNA binders), a drastic reduction in the number of nonspecific interactions was observed if a PLL-*g*-PEG-coated surface was incubated overnight in PBS. This prompted an investigation into the effect of overnight PBS incubation, before the ssDNA conjugation, on the molecular distribution of PLL-*g*-PEG-ssDNA molecules. Indeed, we observed a more homogeneous (lower distribution score, indicating less dispersion) biofunctionalized surface when the PLL-*g*-PEG-coated surface is incubated overnight in PBS prior to ssDNA conjugation.

It is clear that the biofunctionalization conditions play a role in the spatial heterogeneity of the surface-bound binder molecules. Based on the observations, we hypothesize three molecular mechanisms that may occur during the biofunctionalization process and affect the spatial properties of surface-bound ssDNA binders. After PLL-*g*-PEG adsorption, the following could occur:

1. SPAAC click reaction: DBCO-functionalized ssDNA binders approach the azide functional end-group of the PLL-*g*-PEG and form a covalent bond, conjugating the ssDNA binders to the low-fouling PLL-*g*-PEG layer.
2. PLL-*g*-PEG rearrangement: PLL-*g*-PEG molecules are immobilized on negatively charged surfaces via electrostatic interaction. When the polymer solution is removed and exchanged with the binder solution, loosely bound polymer molecules could desorb from the surface, revealing patches of the underlying substrate. It is then favorable for the still-bound polymer molecules to rearrange over the empty patches to form an equilibrium conformation and molecular structure that has fewer loops and tails. This process is evident in Figure 5B(i) and Supplementary Figure S12 when only PLL-*g*-PEG molecules were immobilized. The fact that fewer interaction sites were observed when the substrate is incubated overnight in PBS is in agreement with the hypothesis that the PLL-*g*-PEG molecules might adopt a molecular conformation and structure with well-extended PEG side chains (and fewer loops and tails), resulting in a low-fouling surface.
3. DBCO-ssDNA interacting with lysine in PLL-*g*-PEG: Instead of desorbing from the surface, some of the loosely bound polymer molecules may stay at the surface while some of their positively charged lysine groups are exposed and can interact with negatively charged ssDNA in the solution. In this case, the ssDNA binders are nonspecifically coupled to the surface. The electrostatic interactions between the ssDNAs and lysine groups are also dependent on the salt content of the buffer as detailed in Supplementary Section 4.

Without the additional incubation step, all three scenarios could happen at the same time, and they could compete with each other. We hypothesized that the ionic strength of the conjugation buffer may play a role in enhancing or suppressing certain mechanisms, see Supplementary Section 4. By incorporating a PBS overnight incubation step prior to ssDNA conjugation, Mechanism 3 could be strongly reduced. Substrates prepared with this condition have less dispersed PLL-*g*-PEG-ssDNA molecules, resulting in a more homogeneous biofunctionalized surface.

## 3 Conclusions

We described a single-molecule characterization method based on DNA-PAINT to analyze the spatial molecular properties of high-density biofunctionalized surfaces. Three analysis methods were established to extract the molecular density of PLL-*g*-PEG-ssDNA molecules and ssDNA affinity binder molecules. On top of that, lifetime and binder undersampling were studied and compensated in the analysis using simulation models. From the extracted spatial locations of the PLL-*g*-PEG-ssDNA molecules, we also quantified their spatial distribution via the Clark-Evans test. To demonstrate the capabilities of the analysis framework, we showed that high binder molecule densities of up to 1000 molecules μm^–2^ can be quantified using a relatively short 1-h DNA-PAINT measurement. From the analysis, we conclude that the spatial molecular heterogeneity varies with the various biofunctionalization conditions. We found an increasing molecular dispersion for increasing bioconjugation duration, while the molecular dispersion can be reduced via an additional incubation step. The analysis sheds light on the molecular mechanisms that could occur during the biofunctionalization process.

The described methodology addresses the fundamental challenge of characterizing high-density surface-coupled biomolecules. A recent study by Riera et al. showed that a qPAINT-based counting approach can quantify densities up to several hundred molecules per square micrometer, but loses the capability to study the nanoscale organization of the biomolecules.^10^ On the other hand, the resolution enhancement by sequential imaging (RESI) methodology^11^ has an advantage in resolving individual biomolecules within a DNA-PAINT resolution-limited area, providing information on the molecular distribution of individual binder molecules. However, many imaging rounds and labels (and thus long acquisition times) are necessary to characterize high-density sensor surfaces, and the acquisition time for one imaging round of RESI is estimated to scale with the square of molecular densities, see Supplementary Section 6.3. This hinders the potential of RESI to be used as a high-throughput characterization technique for biofunctionalization studies. The DNA-PAINT analysis framework developed in this article can quantify high molecular densities and reveal the molecular distribution of biomolecules while using a relatively short acquisition time. We estimate that a DNA-PAINT measurement as short as 5 min would be sufficient for analysis provided that the imager concentration is optimal, i.e., near the theoretical maximum allowed imager concentration, see Supplementary Section 2.

The described analysis methodology paves the way toward utilizing DNA-PAINT as a versatile characterization tool for evaluating biofunctionalization density and heterogeneity in densely functionalized samples. Without modifying the fundamentals of DNA-PAINT imaging, the methodology takes advantage of the single-molecule data obtained in a short time (∼1 h) to quantify the molecular density and the spatial distribution of the biomolecules, enabling the use of these parameters to study the biofunctionalization quality in terms of yield, efficiency, and homogeneity. Since DNA-PAINT has been demonstrated to image and quantify various types of target molecules,^19,28−31^ the described analysis is generalizable to other biofunctionalization strategies, molecular systems, and a variety of surface materials, provided that microscopy data of high quality can be obtained. By labeling the target biomolecule of interest with a docking ssDNA-functionalized labeling probe (e.g., protein G, protein M, nanobody) and imaging the labeling probes using standard DNA-PAINT procedures, the analysis methodology described in this article can be applied to quantify the biomolecule density and spatial molecular distribution.

To conclude, we have demonstrated a robust analysis method based on DNA-PAINT imaging to study the spatial molecular heterogeneity of high-density surface-bound biomolecules. In the future, this method can be extended to study the spatial homogeneity of affinity molecules on synthetic surfaces made of different materials (organic, inorganic, metal, etc.) and different shapes (flat surfaces, textured surfaces, particles, rods, etc.). Combining the versatility of DNA-PAINT imaging and the described analysis, we foresee the potential of DNA-PAINT as a single-molecule characterization technique to evaluate the biofunctionalization quality and guide future biofunctionalization strategies for a wide range of applications, including bioactive implantables, regenerative medicine, targeted drug delivery, and bioanalysis.

## 4 Experimental Section

### 4.1 Materials and Chemicals

Glass coverslips (22 × 40 mm, thickness #1.5, Epredia) were obtained from VWR. Custom-made flow cell stickers with an approximate internal volume of 20 μL were obtained from Grace Biolabs (USA). Poly(l-lysine)-grafted poly(ethylene glycol) (PLL-*g*-PEG) with a grafting ratio of 3.5 was purchased from SuSoS (Switzerland). The molecular weight of the PLL backbone and PEG side chains are 20 and 2 kDa, respectively. Azide functionalized PLL-*g*-PEG (Nanosoft Biotechnology LLC, USA) is composed of a 15 kDa PLL backbone and 2 kDa PEG chain with a grafting ratio of 5. Fluorescent nanoparticles (0.2 μm, yellow-green, Molecular Probes) were used as fiducial markers. PBS tablets, NaCl, and Mg_2_Cl were purchased from Sigma-Aldrich, and Tween 20, EDTA, and Tris-HCl were purchased from Merck Life Science. The ssDNA oligonucleotides (standard desalting and HPLC purification for chemically modified DNA) were purchased from IDT (Integrated DNA Technologies). All ssDNA sequences are detailed in the Supplementary Table S6.

PBS buffer was prepared by dissolving 1 tablet of PBS in 200 mL of Milli-Q water. 1 M of NaCl dissolved in PBS was used as the high-salt (HS) buffer in this study. The imaging buffer (Buffer B) consists of 10 mM Mg_2_Cl, 5 mM Tris-HCL, 1 mM EDTA and 0.05% Tween 20.

### 4.2 Substrate Surface Functionalization

The binder molecule is a partially dsDNA consisting of two complementary ssDNA, namely binder ssDNA and DBCO-functionalized ssDNA. The binder molecules were always prehybridized and used as a stock solution of 10 μM for subsequent surface functionalization. The prehybridization protocol involves mixing a 4:1 molar ratio of binder ssDNA to DBCO-functionalized ssDNA (both at a concentration of 100 μM) in HS buffer on a rotating fin for at least 2.5 h.

The coverslips were washed by 10 min of sonication in isopropanol and Milli-Q baths, respectively. Polymer mixture solution, consisting of 1% v/v PLL-*g*-PEG-N3/PLL-*g*-PEG unless stated otherwise, is prepared from stock solutions of PLL-*g*-PEG (1 mg/mL) and PLL-*g*-PEG-N3 (1 mg/mL) and diluted with Milli-Q to a final combined concentration of 0.5 mg/mL. After the sonication steps, the substrates were dried under nitrogen flow and placed under 1 min of oxygen plasma to oxidize the surface. A flow cell sticker was then attached to the substrate and the polymer mixture solution was immediately added to the flow cell and incubated for approximately 3 h. The solution in the flow cell was then aspirated to remove unbound or loosely bound polymer molecules and replaced with PBS or binder solution (diluted to 2 μM from stock with HS buffer). In the overnight PBS incubation experiment, the buffer in the flow cell was exchanged with the binder solution after overnight incubation. The incubation time of the binder solution was varied in this work.

### 4.3 DNA-PAINT Imaging and Data Analysis

Fiducial markers were prepared by first sonicating the stock solution for 5 min to disaggregate clusters of the particles. After sonication, the stock suspension was diluted 10,000 times with PBS and the diluted suspension was subjected to 5 min sonication. Before flowing in the fiducial markers, the substrates were flushed with 100 μL of PBS to remove unreacted binder molecules and to exchange for fresh buffer. Then, the buffer in the flow chamber was exchanged with the suspension of fiducial markers, and the fiducial markers were allowed to sediment and attach to the substrate for 5 min. After this procedure, the unattached fiducial markers were flushed away with 100 μL of Buffer B, and imager ssDNA solution (diluted in Buffer B) was added into the flow chamber.

DNA-PAINT imaging was performed on Oxford Nanoimager with a TIRF configuration. Fluorescence was recorded using a 100×, 1.4 NA oil immersion objective, passed through a beam splitter to obtain a green and a red channel. Images were acquired with an exposure time of 100 ms under 12 mW of 640 nm laser and 0.05 mW of 532 nm laser illumination simultaneously for 1 h. The concentration of the imager was fixed at 25 pM for all experiments. The imager concentration is tuned using the biofunctionalized surface prepared with 10% v/v PLL-*g*-PEG-N3/PLL-*g*-PEG. By evaluating the quality of the microscopy images (ensuring minimal overlap of PSFs), an imager concentration of 25 pM was found to be optimal and was kept constant for all experiments to allow comparisons between different bioconjugation conditions.

The raw images were then analyzed with ThunderSTORM,^32^ an open-source plug-in in ImageJ, to extract the localizations of the fluorescence emissions. Afterward, the localizations and their properties were used as inputs in a custom-written Python script for density and distribution analysis. The *p*-values provided in this work were obtained via Welch’s *t*-test statistic.

## Supporting Information Available

Information on the molecular system employed, DNA-PAINT experimental requirements for densely functionalized surfaces, details on the developed data analysis [direct counting analysis, kinetic counting, compensation for lifetime undersampling analysis, and Clark-Evans test], ionic strength dependence of the spatial molecular heterogeneity, dispersion of PLL-*g*-PEG-ssDNA molecules, and additional information and extended data on ssDNA sequences used, nonspecific interactions for control surfaces, and application of resolution enhancement by sequential imaging for densely functionalized sample (PDF)
