# Supplementary material for: Revealing Spatial Molecular Heterogeneity of High-Density Biofunctionalized Surfaces Using DNA-PAINT
Source: ACS Appl Mater Interfaces. 2024 Oct 21;16(43):58191–202. doi: 10.1021/acsami.4c10310 (PMC11533166; doi:10.1021/acsami.4c10310)
Supplement: Supplementary file 1 — am4c10310_si_001.pdf [file am4c10310_si_001.pdf]

Supporting Information: Revealing spatial molecular heterogeneity of high-density biofunctionalized surfaces using DNA-PAINT

Wei Shan Tan,[†,‡] Arthur M. de Jong,[¶,‡] and Menno W. J. Prins[*,†,¶,‡,§]

†*Department of Biomedical Engineering, Eindhoven University of Technology, Eindhoven, 5612 AZ, The Netherlands*

‡*Institute for Complex Molecular Systems (ICMS), Eindhoven University of Technology, Eindhoven, 5612 AZ, The Netherlands*

¶*Department of Applied Physics, Eindhoven University of Technology, Eindhoven, 5612 AZ, The Netherlands*

§*Helia Biomonitoring, Eindhoven, 5612 AZ, The Netherlands*

E-mail: m.w.j.prins@tue.nl

Contents

1 Molecular system **S-3**

2 DNA-PAINT experimental requirements for densely-functionalized surfaces **S-9**

3 Data analysis **S-12**

3.1 Direct counting (DC) analysis S-12

3.2 Kinetic counting (KC) and Compensation for Lifetime Undersampling (CLiU) analysis S-15

3.3 Compensation for Binder Undersampling (CBiU) analysis S-19

3.4 Clark-Evans test S-22

4 Ionic strength dependence of the spatial molecular heterogeneity **S-26**

5 Dispersion of PLL-g-PEG-ssDNA molecules **S-29**

6 Additional information and extended data **S-32**

6.1 ssDNA sequences S-32

6.2 Investigation of non-specific interactions for control surfaces S-33

6.3 Application of Resolution Enhancement by Sequential Imaging for densely-functionalized sample S-35

References **S-40**

1 Molecular system

The model system in this study involves the use of poly(l-lysine)-graft-poly(ethylene glycol) (PLL-*g*-PEG) coating and coupling of single-stranded DNA (ssDNA) binders on the low-fouling coating via click chemistry. A mixture of PLL-g-PEG molecules and azide-functionalized PLL-g-PEG (PLL-g-PEG-N_3) are first coated on a glass surface. Thereafter the dibenzocyclooctyne-functionalized ssDNA (DBCO-ssDNA) binders are conjugated to the polymer layer via strain-promoted azide-alkyne cycloaddition (SPAAC) click reaction.

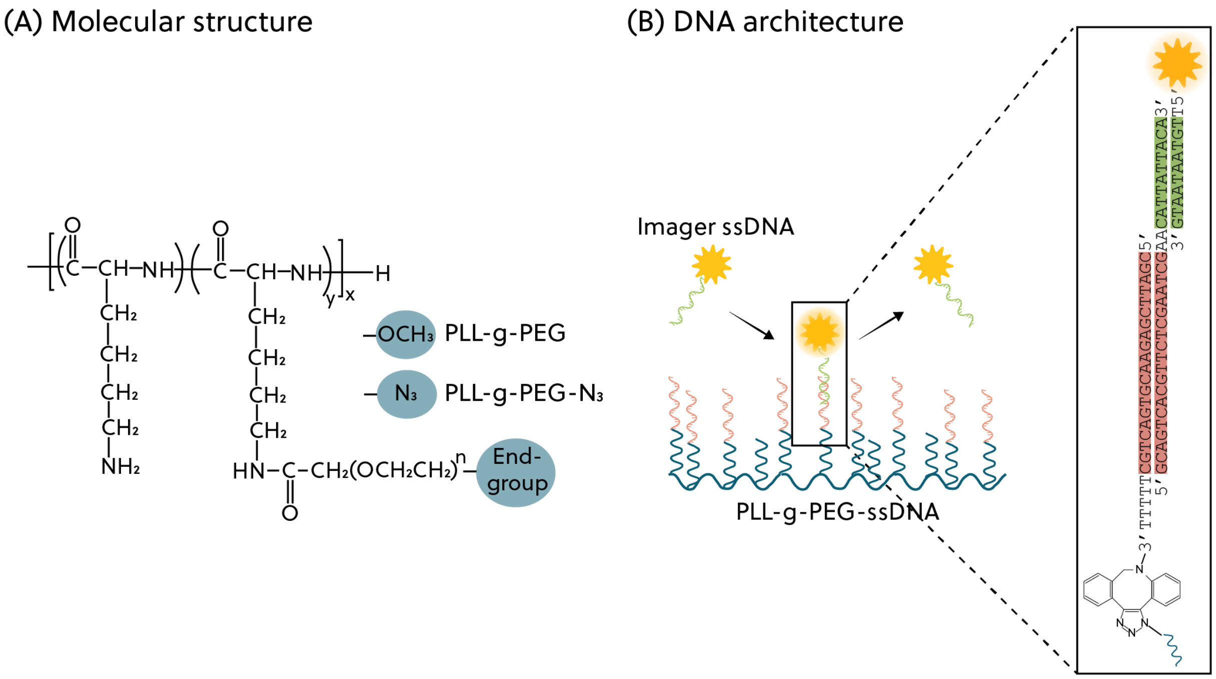

Figure S1: (A) Molecular structure of PLL-g-PEG molecule and PLL-g-PEG-N_3 molecule. (B) DNA architecture used in this study.

The molecular structure and architecture of the PLL-g-PEG and PLL-g-PEG-ssDNA molecules are sketched in Fig. S1A. For the PLL-g-PEG molecules with similar PEG length and grafting ratio as in this work, studies have shown that the PLL backbones of the PLL-g-PEG molecules adsorb on negatively charged glass surfaces and the PEG side chains are extended perpendicular from the surface and into the solution.[S1,S2] From this, we estimate

the polymer coverage density $\sigma_{\mathrm{polymer}}$ on a surface to be given by,

$$\sigma_{\mathrm{polymer}} = \frac{1}{A_{\mathrm{polymer}}} = \frac{1}{l_{\mathrm{backbone}} \cdot d_{\mathrm{interchain}}} \tag{S1}$$

where the A_{polymer}, l_{backbone} and $d_{\mathrm{interchain}}$ denote the area of the PLL backbone, PLL backbone contour length and the interchain distance between neighboring PLL-g-PEG molecules. l_{backbone} is computed by considering the bond length between carbon atoms $l_{\mathrm{C-C}}$ (0.154 nm), the bond length between carbon and nitrogen atoms $l_{\mathrm{C-N}}$ (0.143 nm), the bond angle between carbon atoms $\theta_{\mathrm{C-C}}$ (120°), the bond angle between carbon and nitrogen atoms $\theta_{\mathrm{C-N}}$ (109.5°), and the number of lysine monomers n_{lys} in the PLL backbone,

$$l_{\mathrm{backbone}} = \left(l_{\mathrm{C-C}} \sin\left(\frac{\theta_{\mathrm{C-C}}}{2}\right) + 2 l_{\mathrm{C-N}} \sin\left(\frac{\theta_{\mathrm{C-N}}}{2}\right) \right) n_{\mathrm{lys}}. \tag{S2}$$

This equation assumes that the PLL-g-PEG molecules are well-extended on the surface.

However, since we coat a mixture of PLL-g-PEG (denoted by PLL) and PLL-g-PEG-N_3 (denoted by PLL, N_3) on the surface, the mixing ratio r_{m} (mass concentration ratio of PLL-g-PEG-N_3 to total polymer concentration) plays a role in estimating the PLL-g-PEG-N_3 coverage density $\sigma_{\mathrm{PLL,\ N_3}}$ on the surface. Firstly, the mixing ratio in terms of mass concentration is first converted to the mixing ratio in terms of number ratio r_{n} via,

$$\begin{aligned}
r_{\mathrm{n}} &= \frac{n_{\mathrm{PLL,\ N_3}}}{n_{\mathrm{PLL}} + n_{\mathrm{PLL,\ N_3}}} \\
&= \frac{c^{\mathrm{mol}}_{\mathrm{PLL,\ N_3}}}{c^{\mathrm{mol}}_{\mathrm{PLL}} + c^{\mathrm{mol}}_{\mathrm{PLL,\ N_3}}} \\
&= \frac{c^{\mathrm{mass}}_{\mathrm{PLL,\ N_3}}/M_{\mathrm{PLL,\ N_3}}}{c^{\mathrm{mass}}_{\mathrm{PLL}}/M_{\mathrm{PLL}} + c^{\mathrm{mass}}_{\mathrm{PLL,\ N_3}}/M_{\mathrm{PLL,\ N_3}}} \\
\text{Given that } r_{\mathrm{m}} &= \frac{c^{\mathrm{mass}}_{\mathrm{PLL,\ N_3}}}{c^{\mathrm{mass}}_{\mathrm{tot}}}, \\
r_{\mathrm{n}} &= \frac{r_{\mathrm{m}} c^{\mathrm{mass}}_{\mathrm{tot}}}{(1 - r_{\mathrm{m}}) c^{\mathrm{mass}}_{\mathrm{tot}} \frac{M_{\mathrm{PLL,\ N_3}}}{M_{\mathrm{PLL}}} + r_{\mathrm{m}} c^{\mathrm{mass}}_{\mathrm{tot}}}
\end{aligned} \tag{S3}$$

where n_x denotes the number of polymer x molecules, $c_\mathrm{x}^\mathrm{mol}$ the molar concentration of polymer x, $c_\mathrm{x}^\mathrm{mass}$ the mass concentration of polymer x, M_x the molar weight of polymer x, and $c_\mathrm{tot}^\mathrm{mass}$ the total polymer mass concentration. With this quantity, the coverage density of PLL-g-PEG-N_3 $\sigma_{\mathrm{PLL,\ N_3}}$can be computed,

$$\sigma_\mathrm{tot} = \frac{1}{(1-r_\mathrm{n})A_\mathrm{PLL} + r_\mathrm{n}A_{\mathrm{PLL,\ N_3}}}$$

$$\text{Given that } r_\mathrm{n} = \frac{n_{\mathrm{PLL,\ N_3}}}{n_\mathrm{tot}},$$

$$\sigma_{\mathrm{PLL,\ N_3}} = \frac{r_\mathrm{n}}{(1-r_\mathrm{n})A_\mathrm{PLL} + r_\mathrm{n}A_{\mathrm{PLL,\ N_3}}} \tag{S4}$$

where σ_tot denotes the total polymer coverage density.

Table S1: Properties of PLL-g-PEG and PLL-g-PEG-N_3 molecules used in this work.

		PLL-g-PEG	PLL-g-PEG-N_3
Molar weight of PLL backbone	(g mol^{-1})	24700	15000
Graft ratio $\frac{n_\mathrm{lys}}{n_\mathrm{PEG}}$	(-)	3.5	5
Molar weight of PEG chain	(g mol^{-1})	2164	2000
Average number of lysine monomer n_lys	(-)	169	103
Average number of PEG side chains n_PEG	(-)	48	21
PLL backbone contour length l_backbone	(nm)	62	38
Interchain distance $d_\mathrm{interchain}$ [S3]	(nm)	2	2
PLL backbone area A	(nm^2)	124	75
Molar weight of polymer M	(g mol^{-1})	129160	56042

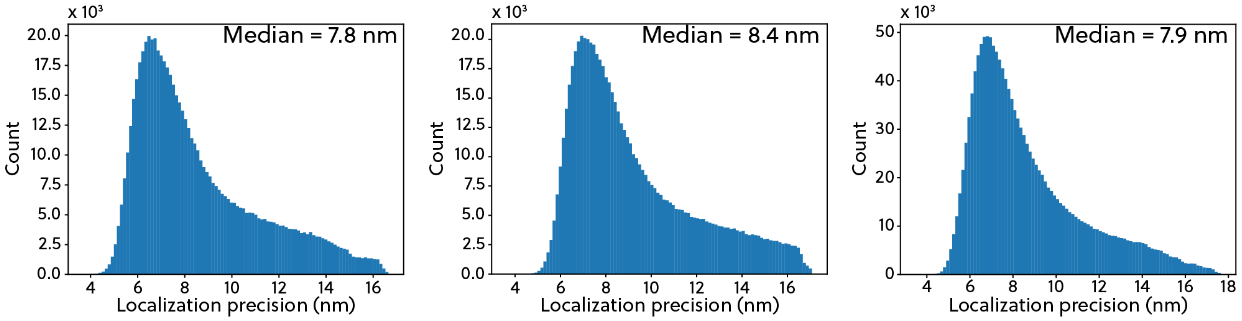

Figure S2: The average localization precision of DNA-PAINT measurement is quantified to be approximately 8 nm.

For $c_\mathrm{tot}^\mathrm{mass}$ = 0.5 mg/mL and r_m = 0.01 or 0.1, the estimated PLL-g-PEG-N_3 density

$\sigma_{\mathrm{PLL,\ N_3}}$ is found to be $185\,\mu\mathrm{m}^{-2}$ and $1787\,\mu\mathrm{m}^{-2}$ respectively. Provided that each PLL-g-PEG-N_3 molecule is coupled to at least one ssDNA binder and thus can be imaged using DNA-PAINT, the expected nearest neighbor distance between each PLL-g-PEG-ssDNA molecule $\mathrm{E}(d_{\mathrm{PLL-g-PEG-ssDNA}})$ can be estimated by,

$$\mathrm{E}(d_{\mathrm{PLL-g-PEG-ssDNA}}) = \frac{1}{2\sqrt{\sigma_{\mathrm{PLL-g-PEG-ssDNA}}}}. \tag{S5}$$

This corresponds to $\mathrm{E}(d_{\mathrm{PLL-g-PEG-ssDNA}})$ values of 37 nm and 12 nm for 1% and 10% mixing ratio. Since the average localization precision of DNA-PAINT measurement is quantified to be approximately 8 nm (see Fig. S2), we expect that imaging the PLL-g-PEG-ssDNA molecules is still within the resolution limit of DNA-PAINT and the density of localization clouds as quantified in the direct counting (DC) analysis can be interpreted as the density of PLL-g-PEG-ssDNA molecules.

To estimate the binder density on the surface, we need to consider the geometry of the binder molecule and how many binder molecules can fit onto one PLL-g-PEG-N_3 molecule. To start with, the binder molecule is a partially double-stranded DNA with 12 free bases extending into the solution, see Fig. S1. Due to this partially double-stranded architecture, we approximate the width of the binder molecule by the width of a DNA double-helix structure ($\approx 2\,\mathrm{nm}$).[S4] The average distance between PEG-N_3 side chains d_{PEG} is given by

$$d_{\mathrm{PEG}} = \frac{l_{\mathrm{backbone,\ PLL,\ N_3}}}{n_{\mathrm{PEG}}} \tag{S6}$$

and is calculated to be 1.8 nm. Since the width of the binder molecule is slightly larger than the average distance between PEG-N_3 side chains, we estimate that 10 binder molecules can be coupled to PEG-N_3 side chains at the maximum. Keeping this in mind, we estimate the binder density σ_{ssDNA} on the surface to be approximately $1.85 \times 10^3\,\mu\mathrm{m}^{-2}$ and $1.79 \times 10^4\,\mu\mathrm{m}^{-2}$ for a mixing ratio of 1% and 10% respectively.

Furthermore, to perform a temporal analysis of the obtained DNA-PAINT localization,

Table S2: The estimated values to study the DNA hybridization process in DNA-PAINT.

		1% mixing ratio	10% mixing ratio
σ_{ssDNA}	(μm^{-2})	1851	17874
h_{chamber}	(μm)	450	450
k_{off}	(s^{-1})	1	1
Estimated k_{on}	(M^{-1} s^{-1})	10^6	10^6
c_{binder}	(M)	6.8×10^{-9}	6.6×10^{-8}
c_{img}	(M)	2.5×10^{-11}	2.5×10^{-11}
K_{d}	(M)	10^{-6}	10^{-6}

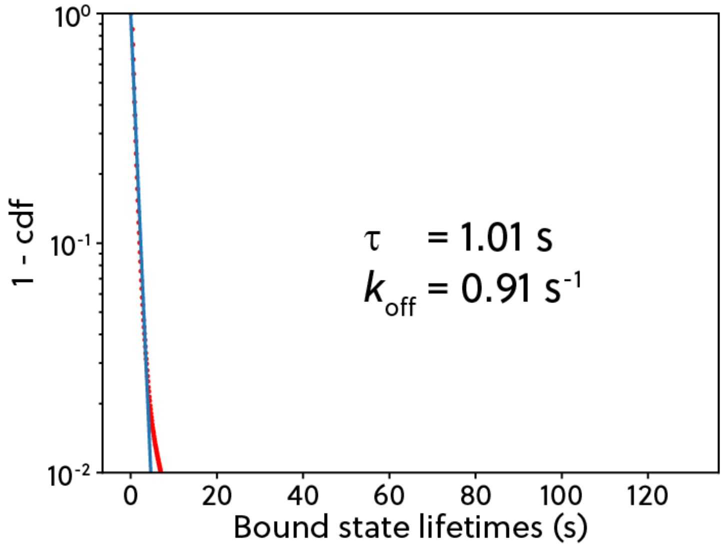

Figure S3: The experimental k_{off} value is quantified as $0.91\,\mathrm{s}^{-1}$. For the Monte Carlo simulation, the k_{off} value is approximated as 1.

the molecular picture of the DNA hybridization process needs to be estimated, i.e., whether the DNA hybridization is governed by the ssDNA binder excess regime or the imager ssDNA excess regime. Given the estimated $\sigma_{\mathrm{PLL,\ N_3}}$, the effective volumetric binder concentration is computed by

$$c_{\mathrm{binder}} = \frac{\sigma_{\mathrm{ssDNA}}}{1000 \cdot N_{\mathrm{A}} \cdot h_{\mathrm{chamber}}} \tag{S7}$$

where N_{A} denotes Avogadro's number and h_{chamber} the height of the measurement chamber (in this work, the height of the flow chamber is approximately $450\,\mu$m).

The equilibrium dissociation constant K_{d} indicates the interaction affinity between the

ssDNA binders and the imager ssDNA strands and is calculated using

$$K_{\mathrm{d}} = \frac{k_{\mathrm{off}}}{k_{\mathrm{on}}} \tag{S8}$$

where k_{off} and k_{on} denote the molecular dissociation rate and association rate. The dissociation rate is obtained from experimental data as seen in Fig. S3, while the association rate is assumed to be $10^6\,\mathrm{M}^{-1}\mathrm{s}^{-1}$, equivalent to the association rate of a well-defined single ssDNA docking strand such as on DNA origami. Given these values, we expect the DNA hybridization kinetics to be governed by the binder excess regime since $c_{\mathrm{binder}} \gg c_{\mathrm{img}}$.

2 DNA-PAINT experimental requirements for densely-functionalized surfaces

Due to the high-density of biomolecules functionalized on the surface, it is crucial to tune the imager concentration such that the raw fluorescence images are not saturated with fluorescent emissions. To estimate the theoretical maximum imager concentration that can be used, we calculate the minimum distance between point spread functions of the emitters $d_{\mathrm{PSF,min}}$ according to the Rayleigh criterion:

$$d_{\mathrm{PSF,min}} = 0.61 \times \frac{\lambda}{\mathrm{NA}} \tag{S9}$$

where λ denotes the emission wavelength of the fluorophores (in this article, $\lambda = 664\,\mathrm{nm}$ for the dye ATTO647N) and NA denotes the numerical aperture of the objective lens (NA = 1.4). If the distance between two point spread functions falls below this minimum distance $d_{\mathrm{PSF,min}}$, the emitters spatially overlap and can no longer be precisely localized.

Given this minimum distance, we estimate the maximum density of emitters that are allowed to be in a single frame of measurement $\sigma_{\mathrm{PSF,max}}$ without causing spatial overlap of emitters to be $3.0\,\mu\mathrm{m}^{-2}$ *via*

$$\sigma_{\mathrm{PSF,max}} = \left(\frac{1}{2 \times d_{\mathrm{PSF,min}}}\right)^2 . \tag{S10}$$

This relation takes into account the stochastic nature of the fluorescent emissions.

This maximum emitter density can also be interpreted as the maximum number of binding events that is allowed to be observed at any given point in time (in one frame of measurement), and is related to the kinetic parameters of the molecular system. By considering the probability that the ssDNA binders are being visited and bound to an imager strand, we find the relation between theoretical maximum imager concentration $c_{\mathrm{img,max}}$ and the molecular

density σ_{mol} to be

$$\mathrm{P(bound)} = \frac{c_{\mathrm{img}}}{c_{\mathrm{binder}} + K_{\mathrm{d}}} \simeq \frac{c_{\mathrm{img}}}{K_{\mathrm{d}}} \qquad \mathrm{P(bound)} = \frac{n_{\mathrm{PSF,max}}}{n_{\mathrm{mol}}} = \frac{\sigma_{\mathrm{PSF,max}}}{\sigma_{\mathrm{mol}}}$$

$$c_{\mathrm{img,max}} = K_{\mathrm{d}} \times \frac{\sigma_{\mathrm{PSF,max}}}{\sigma_{\mathrm{mol}}} \tag{S11}$$

where c_{binder} is the effective volumetric binder concentration and K_{d} is the equilibrium dissociation constant (see equation S8). This relation assumes that the DNA hybridization kinetics is governed by the binder excess regime. Given that K_{d} is estimated to be 1 µM, the theoretical maximum imager concentration is found to range from nM to pM for molecular densities ranging from $10^2\,\mu\mathrm{m}^{-2}$ to $10^5\,\mu\mathrm{m}^{-2}$.

To estimate the acquisition time required, we have to consider the foundation on which the analysis framework in this article is based. In this work, we utilized a direct counting approach to obtain the density of localization clouds on the surface. Riera et al. demonstrated that such direct counting approach relies on identifying individual localization clouds on a given area, and no longer provides a reliable count when these localization clouds spatially overlap in the time-aggregated localization plot.[S5] This means that there is a maximum density of localization clouds $\sigma_{\mathrm{cloud,max}}$ that are allowed to be observed in a single experiment. We approximate this value *via*

$$\sigma_{\mathrm{cloud,max}} = \left(\frac{1}{2 \times a_{\mathrm{search}}}\right)^2 \tag{S12}$$

where a_{search} denotes the search region or bandwidth employed in the direct counting analysis. This relation assumes that the localization clouds are distributed in a random fashion. Given the search region a_{search} in this work is approximately 30 nm, the maximum density of localization clouds allowed $\sigma_{\mathrm{cloud,max}}$ is $277\,\mu\mathrm{m}^{-2}$.

The total acquisition time is related to the DNA hybridization kinetics (thus to the molecular density σ_{mol} and the imager concentration c_{img}), and must adhere to the require-

ments stated above. By performing simple Monte Carlo simulations to simulate binding events based on a given molecular density and theoretical maximum imager concentration $c_{\mathrm{img,max}}$ for the given density, we evaluate the acquisition duration that gives a total number of events less than the maximum density of localization clouds allowed $\sigma_{\mathrm{cloud,max}}$. From the simulations, we found that the total acquisition duration can be as short as 5 min for molecular densities ranging from $10^2\,\mu\mathrm{m}^{-2}$ to $10^5\,\mu\mathrm{m}^{-2}$ if the theoretical maximum imager concentration $c_{\mathrm{img,max}}$ is employed.

3 Data analysis

3.1 Direct counting (DC) analysis

The DC analysis relies on a mean-shift clustering algorithm to identify the localization clouds in the DNA-PAINT localization data. Mean-shift analysis is a non-parametric, density estimation-based feature-space analysis.[S6–S8] This analysis regards the feature space (in this case, the time-aggregated localization plot) as the empirical probability density function of the represented parameter (spatial locations of localization clouds). Thus, dense regions in feature space, i.e., clouds of localizations in the localization plot, correspond to the modes of the unknown density. Once the mode is found, the cluster (the localizations) associated with it is determined based on the local structure of the feature space and the bandwidth parameter that relates to the size of the dense regions. The full implementation of the mean-shift procedure is detailed in Comanicìu and Meer.[S8]

The advantages of mean-shift analysis lie in the fact that it does not require a priori knowledge of the number of clusters (number of localization clouds) present and it does not assume the same shape for all the clusters in space. The flexibility of the analysis allows it to be applied not only to DNA-PAINT imaging data of a flat substrate but also to data of varying structures (e.g., spherical particle, rod-like structure). Although the mean-shift analysis is not computationally expensive, it does not scale well with the dimension of space and it is not recommended for analyzing feature space with more than 6 dimensions.[S8] Considering that the DNA-PAINT localization data used for DC analysis consists of only two dimensions (x- and y-coordinates of the localizations), the mean-shift algorithm is more than adequate to find the spatial locations of the localization clouds.

This work utilizes the implementation of mean-shift analysis in Scikit-learn.[S9] To investigate the effect of the bandwidth parameter on the analysis result, localization clouds with a given size were simulated, and they were used as inputs in the mean-shift algorithm with varying bandwidth values. Fig. S4A shows two examples where the bandwidth is set too

low or too high. If the bandwidth is set too low, one localization cloud is identified as two or more localization clouds, resulting in more identified localization clouds than the true value. On the contrary, for a bandwidth that is set too high, multiple localization clouds that are close to each other are identified just as one, causing an underestimation of the number of localization clouds. Therefore, there is a sweet spot in the bandwidth parameter selection to obtain the most accurate result from the mean-shift analysis, as seen in Fig. S4(B).

For experimental data, we do not know the true bandwidth or size of the localization cloud. To ensure the reliability of the analysis, we consider the localization precision of the localization data and approximate the bandwidth to be twice the maximum xy-localization uncertainty. This means that the size of the localization is dependent on how precise each fluorescent emission can be localized. Similar to the simulated dataset, overestimation (blue circle in Fig. S4C) and underestimation (black circle in Fig. S4C) of the number of found localization clouds can be seen by changing the bandwidth value.

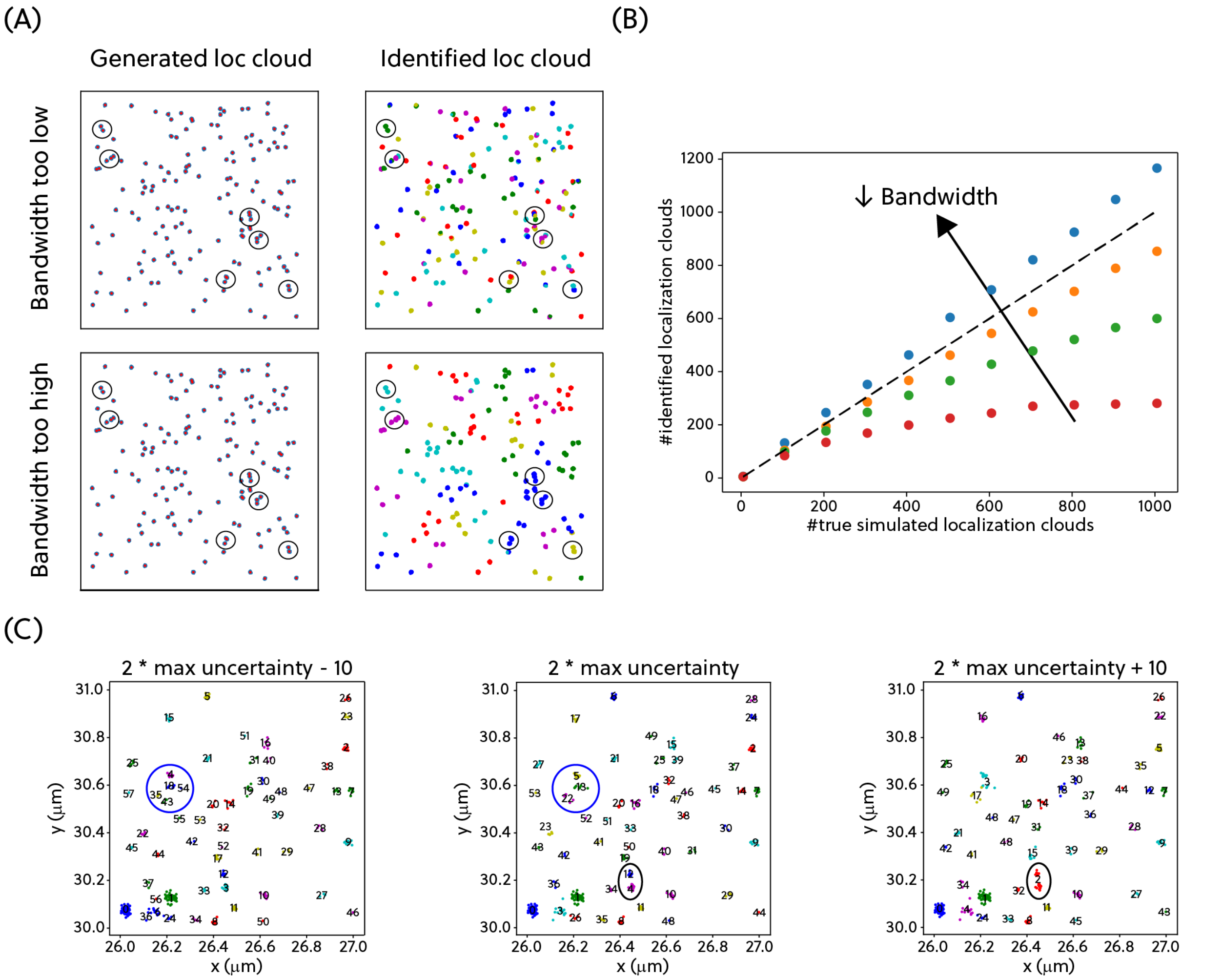

Figure S4: (A) Simulated localization data were used as inputs to the mean-shift analysis with varying bandwidth values. On the left panel, the blue points indicate the localization data while the red points indicate the center of the localization cloud generated. On the right panel, localization clouds are plotted as data with different colors. The black circles show the reference localization clouds to indicate the overestimation and underestimation of the identified localization cloud from different bandwidth values. (B) Different numbers of localization clouds were simulated and used as inputs to the mean-shift analysis with varying bandwidth values. The black dotted line is a guide to the eye to show the ideal analysis result. There is an optimal bandwidth parameter for a given number of simulated localization clouds. (C) Experimental data was analyzed with three bandwidth values to illustrate the effect of bandwidth selection in the identification of the localization cloud.

3.2 Kinetic counting (KC) and Compensation for Lifetime Undersampling (CLiU) analysis

The KC analysis considers the temporal information in each DC-identified localization cloud and aims to obtain an average number of origami-equivalent ssDNA binder per PLL-g-PEG-ssDNA molecule. To investigate the effect of lifetime undersampling on the quantification, Monte Carlo simulations were performed to simulate the signal time traces of each localization cloud in 1 hour.

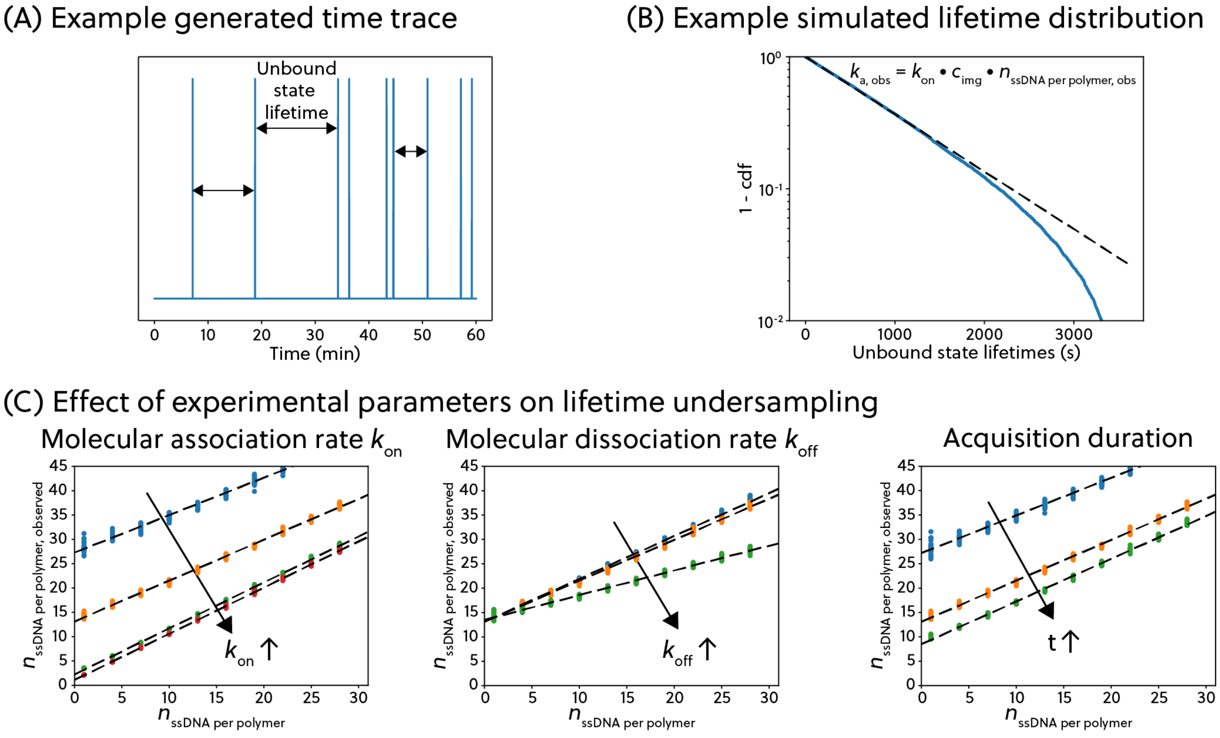

Figure S5: (A) Example time trace that was generated during the simulation. (B) Example distribution of unbound state lifetimes plotted as survival curve (1 - cdf, where cdf denotes cumulative distribution function). The observed association rate $k_{\text{a, obs}}$ is used to calculate the average number of origami-equivalent ssDNA per localization cloud $n_{\text{ssDNA per polymer, obs}}$. (C) Effect of experimental parameters, such as molecular association rate, dissociation rate, and acquisition duration, on the extent of lifetime undersampling.

The time trace of a localization cloud consists of bound state lifetimes (the time duration in which a fluorescent signal is observed) and unbound state lifetimes (the time duration in which no fluorescent signal is observed). The lifetimes are related to the DNA molecular

association rate k_{on} and the dissociation rate k_{off} via

$$\tau_{\text{b}} = \frac{1}{k_{\text{off}}} \qquad \tau_{\text{ub}} = \frac{1}{k_{\text{on}} \cdot c_{\text{img}} \cdot n_{\text{ssDNA per polymer}}} \tag{S13}$$

where τ_{b} denotes the characteristic bound state lifetime, τ_{ub} the characteristic unbound state lifetime, c_{img} the concentration of the imager strand, and $n_{\text{ssDNA per polymer}}$ the number of ssDNA binders per localization cloud. The state lifetimes are generated by sampling from a single-exponential distribution using the characteristic state lifetime.

k_{off} is determined experimentally to be $1\,\text{s}^{-1}$ while a k_{on} reference value, quantified based on DNA origami, is assumed to be $10^6\,\text{M}^{-1}\text{s}^{-1}$.[S10,S11] The k_{on} is not determined in this study because it is not possible to obtain a reliable determination of the value. This is because we lack control in the biofunctionalization process to reliably functionalize each azide-functionalized PLL-g-PEG molecule with only one ssDNA binder. Moreover, it is crucial to note that the association rate is greatly dependent on the accessibility of the binding partner. For a high-density sample, the accessibility of the ssDNA binders can be hindered by neighboring ssDNAs, which may cause a decrease in k_{on}.[S12] Since studying the accessibility of the ssDNA molecules is beyond the scope of this work, a reference value based on DNA-origami is assumed and the impact of association rate on the density quantification is discussed later in this section.

Furthermore, there is a probability that the ssDNA binders in each localization cloud are being visited and bound to an imager strand,

$$\text{P(bound)} = \frac{c_{\text{img}}}{c_{\text{binder}} + K_{\text{d}}} \simeq \frac{c_{\text{img}}}{K_{\text{d}}} \qquad \text{with } K_{\text{d}} = \frac{k_{\text{off}}}{k_{\text{on}}} \tag{S14}$$

where c_{binder} and K_{d} denote the effective volumetric binder concentration and origami-equivalent equilibrium dissociation constant respectively. The equation is valid given that the ssDNA binders are in excess as compared to the imager strands and that the equilibrium dissociation constant is much larger than the binder and imager concentration

($c_{\text{binder}} \gg c_{\text{img}}$, $K_{\text{d}} \gg c_{\text{binder}}$, $K_{\text{d}} \gg c_{\text{img}}$). With these relations, time traces of the localization cloud are generated with 100 ms integration time.

The unbound state lifetimes are extracted from each localization cloud and pooled to plot the cumulative distribution function (cdf) of the lifetimes, as seen in Fig. S5A and Fig. S5B. Fitting a single-exponential equation on the cdf plot, we obtain the observed association rate $k_{\text{a, obs}}$ which is used to compute the observed average number of ssDNA binders per localization cloud $n_{\text{ssDNA per polymer, obs}}$,

$$n_{\text{ssDNA per polymer, obs}} = \frac{k_{\text{a, obs}}}{k_{\text{on}} \cdot c_{\text{img}}}. \tag{S15}$$

The simulation is repeated 20 times for each $n_{\text{ssDNA per polymer}}$ value. The relation between $n_{\text{ssDNA per polymer, obs}}$ and $n_{\text{ssDNA per polymer}}$ is then used to compensate for the lifetime undersampling in experimental data. The simulation parameters are summarized in Table S3.

Table S3: Simulation parameters for CLiU analysis.

Simulation parameter		Value
k_{off}	(s^{-1})	1
k_{on}	($M^{-1}s^{-1}$)	10^6
c_{img}	(M)	2.5×10^{-11}
$n_{\text{ssDNA per polymer}}$	(-)	1 to 30
Simulation duration	(min)	60
Integration time	(s)	0.1

On top of that, the Monte Carlo simulations are used to study the effect of experimental parameters on the lifetime undersampling. The extent of lifetime undersampling, i.e., the linear relation between $n_{\text{ssDNA per polymer, obs}}$ and $n_{\text{ssDNA per polymer}}$, can be decomposed into two components: the slope and the intercept. The slope is interpreted as how the observed association rate (hence the observed number of ssDNA per localization cloud) changes with respect to the true number of ssDNA per localization cloud. Meanwhile, the intercept is an offset between the observed values and the true values, indicating how far the observed value

deviates from the true value. The closer the slope is to 1 and the intercept is to 0, the less impact of the lifetime undersampling there is on the KC quantification.

By varying the molecular association rate and the acquisition duration, we see that the intercept significantly reduces and the slope remains similar when both parameters increase, as shown in Fig. S5C. This is in agreement with a quantitative DNA-PAINT (qPAINT) study showing that the quantification error can be reduced by increasing the imager influx rate ($k_{\mathrm{on}} \cdot c_{\mathrm{img}}$) or the image acquisition time.[S10] Meanwhile, the effect of the molecular dissociation rate is negligible for low k_{off} values, corresponding to long bound state lifetimes. However, it is notable that the slope decreases for large k_{off} value ($10\,\mathrm{s}^{-1}$). This effect is attributed to the integration time that was used in the simulation. When the τ_{ub} approaches the integration time, the extracted unbound state lifetimes become less reliable. From this finding, we recommend using a shorter camera integration time (higher frame rate) in experiments if the imager strand is designed to have a shorter complementarity (shorter than 10 base pairs) with the docking strand to utilize the KC analysis. Other than that, the effect of imager concentration is not investigated as it contributes equally to the molecular association rate in the equations.

3.3 Compensation for Binder Undersampling (CBiU) analysis

The CBiU analysis is motivated by the observation that more PLL-g-PEG-ssDNA binders are being sampled and imaged as the measurement duration increases. In Fig. S6A, the number of localization clouds found in each subsequent 5-min measurement block are shown for two biofunctionalized surfaces. As measurement duration increases, the number of localization clouds that have been found in previous measurement blocks increases, while the number of localization clouds that were newly found in the current measurement block decreases. Overall, the accumulated total number of localization clouds found in the measurement increases over measurement duration, indicating that more binders are being imaged over time. As we do not observe a plateau in the accumulated total localization cloud at the end of the measurement (1 hour), we hypothesize that not all ssDNA binders have been sampled in the DNA-PAINT measurement.

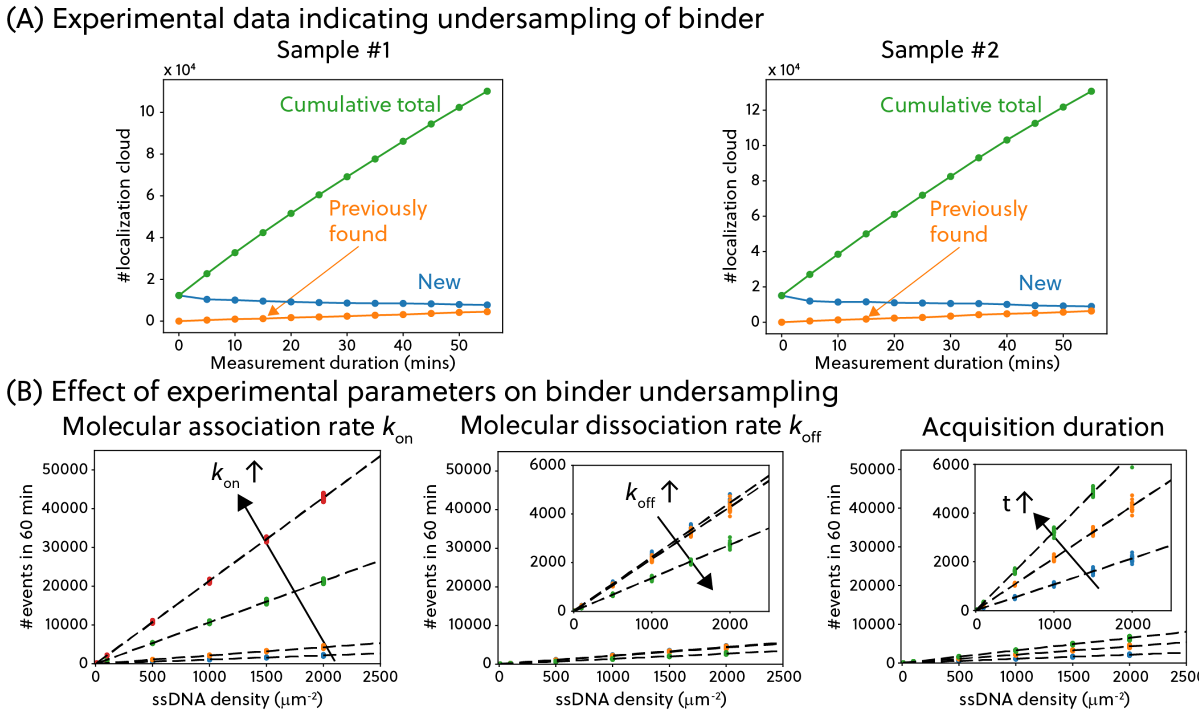

Figure S6: (A) Number of localization clouds found in each subsequent 5-min measurement for two typical biofunctionalized surfaces. (B) Effect of experimental parameters, such as molecular association rate, dissociation rate, and acquisition duration, on the CBiU relation. Insets show the zoomed-in plot of the total number of observed events against the ssDNA binder density.

In the CBiU analysis, Monte Carlo simulations were performed to compensate for the effect of binder undersampling in the quantification of molecular density. In contrast with the simulation set-up for the CLiU analysis, the CBiU analysis simulates signal time traces of each ssDNA binder in a 5×5 µm^2 region of interest in 1 hour. In this case, the characteristic unbound state lifetime τ_{ub} is given by,

$$\tau_{\text{ub}} = \frac{1}{k_{\text{on}} \cdot c_{\text{img}}}. \tag{S16}$$

By adopting the same expression for the characteristic bound state lifetime τ_{b} (equation S13) and the probability of an imager binding to a ssDNA binder (equation S14), and the underlying assumptions, time traces of the individual ssDNA binders are generated with 100 ms integration time. Afterward, the number of binding events observed in each time trace is summed to obtain the total number of observed events for a given region of interest in 1 hour. By varying the ssDNA binder density (hence the number of ssDNA binders), a linear relationship between the ssDNA binder density and the total number of observed events is derived and used to compensate for the binder undersampling in experimental data. The simulation is repeated 20 times for each ssDNA binder density value. The simulation parameters are summarized in Table S5.

Table S4: Simulation parameters for CBiU analysis.

Simulation parameter		Value
k_{off}	(s^{-1})	1
k_{on}	($\text{M}^{-1}\text{s}^{-1}$)	10^6
c_{img}	(M)	2.5×10^{-11}
Simulation duration	(min)	60
Integration time	(s)	0.1
ssDNA binder density	(μm^{-2})	10 to 2000

Furthermore, the Monte Carlo simulations were also used to study how experimental parameters can impact the derived linear relation. The linear relation is described by only a slope component as the intercept is always 0 (zero events are observed when there are no

ssDNA binders). Fig. S6B shows that the molecular association rate has the largest influence over the linear relation. As k_{on} increases, the total number of observed events significantly increases for a given ssDNA density. This is logical because the ssDNA binders are more likely to be bound to the imager strands for higher k_{on}. Since the accessibility of the ssDNA binders for a high-density sample is likely to be hindered, we hypothesize that the actual molecular association rate is on the lower end rather than the opposite.

Albeit not as drastic, the acquisition duration impacts the linear relation similarly to the molecular association rate, with an increasing slope for increasing acquisition duration. This indicates that more binding events between the ssDNA binder and the imager strand can be observed for a longer acquisition duration. On the other hand, the molecular dissociation rate has minimal effect on the total number of observed events. There was a slight decrease in slope for a larger k_{off}; this effect is attributed to the integration time used in the simulation since unbound state lifetimes that are shorter than the integration time are not registered in the time trace as an event.

3.4 Clark-Evans test

Clark and Evans proposed a nearest-neighbor distance-based statistical test to evaluate whether a spatial point pattern is randomly distributed, i.e., the null hypothesis of the complete spatial randomness (CSR) hypothesis.[S13] The test compares the average nearest-neighbor distances (NNDs) of the points to the expected NND and determines whether the observed point pattern is randomly distributed, significantly clustered, or significantly dispersed.

The first thing to consider before constructing the test is the edge effect. Consider that the region of interest is a window through which part of a larger point pattern is being observed, the points that are near the edge of the region of interest will tend to have fewer observed neighbors than points further away from the edge. The observed NND for the points near the edge is then larger than it should be, thus affecting the test statistics and the interpretation of the point distribution.[S14] Therefore, the NNDs of the points lying in the 10% of the edges of the region of interest are not taken into account for analysis.

Following edge removal, the test is constructed by first computing the NNDs of the spatial point pattern (in this work, the DC-quantified spatial location of the PLL-g-PEG-ssDNA molecules). To ensure the validity of the test that assumes independence of NND, a subset of the NNDs of size m d_m^k is randomly chosen and taken for further analysis. With this, the sample mean NND z_m^k can be computed via

$$
\begin{aligned}
\hat{\mu} &= \frac{1}{2\sqrt{\lambda}} \\
\hat{\sigma} &= \sqrt{\frac{4-\pi}{m4\pi\lambda}} \\
z_m^k &= \frac{\overline{d_m}^k - \hat{\mu}}{\hat{\sigma}}
\end{aligned}
\tag{S17}
$$

where λ denotes the point density and is equivalent to $\sigma^{\mathrm{DC}}_{\mathrm{PLL-g-PEG-ssDNA}}$, $\hat{\mu}$ the expected mean NND, $\hat{\sigma}$ the standard deviation of the expected mean NND. Although the effect of

positive dependencies among NNDs has been reduced with the random sub-sampling of NND, the result obtained depends to some degree on the sample selected. To ensure reliable test results, N subsets of the NNDs of size m are randomly selected, such that $k \in [1,\ N]$, and the standardized mean NND $\overline{z_m}$, denoted as the distribution score, can be computed by

$$\overline{z_m} = \frac{\sum^N z_m^k}{N}. \tag{S18}$$

The distribution score is tested against the distribution score of a CSR point pattern at a 5% significance level $z_{0.05} = 1.65$, giving

$$\begin{aligned} \overline{z_m} &< -z_{0.05} \quad : \text{ Significant clustering} \\ \overline{z_m} &> \quad z_{0.05} \quad : \text{ Significant dispersion} \end{aligned} \tag{S19}$$

Table S5: Input parameters for distribution analysis - Clark-Evans test.

Input parameter		Value
Area of region of interest	(μm^2)	25
λ	(μm^{-2})	$\sigma_{\text{DC, PLL-g-PEG-ssDNA}}$
m	(-)	30% of total number of NNDs
N	(-)	1000

To study the effect of undersampling on the distribution quantification, we simulated point patterns with varying point density and degree of clustering, or dispersity. The point patterns are generated such that their mean NND approximates the expected NND (or a factor of the expected NND),

$$\overline{d_m} = a \cdot \hat{\mu} \tag{S20}$$

where a indicates the degree of non-random distribution ($a = 1$ for random point pattern, $a < 1$ for clustered point pattern, and $a > 1$ for dispersed point pattern). Then, we sampled a subset of the generated points and quantified the spatial distribution of the sampled points.

Fig. S7 shows that the undersampling of molecules reduces how significantly the true

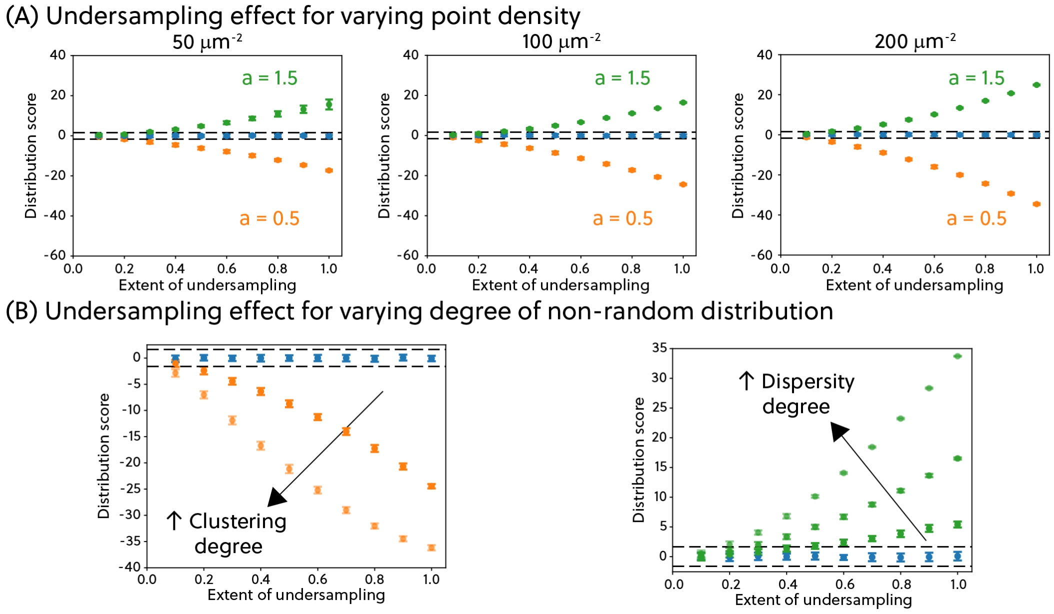

Figure S7: Point patterns of (A) varying point density and (B) varying degrees of non-random distribution are simulated to study the effect of undersampling. In (B), point patterns with a point density of 100 μm^{-2} are simulated.

molecular distribution deviates from the CSR, i.e., the observed distribution score shifts towards zero if fewer molecules are sampled. From the simulation, we found that this effect is more pronounced for point patterns with lower point density, see Fig. S7A. On top of that, if the true molecular distribution is more significantly clustered or dispersed, the observed distribution score is also more resistant to the effect of undersampling. As a rule of thumb, the distribution score quantified in this work should serve as an indication of the true molecular distribution since the issue of undersampling cannot be eliminated due to experimental limitations.

To show that the extent of undersampling does not impact the quantified distribution score in this work, point patterns based on the CBiU-quantified PLL-g-PEG-ssDNA density $\sigma^{\mathrm{CBiU}}_{\mathrm{PLL-g-PEG-ssDNA}}$ and varying degrees of dispersity were simulated, as shown in Fig. S8 (top row). Then, subsets of the generated points were sampled with the experimental sampling ratio ($\frac{\sigma^{\mathrm{DC}}_{\mathrm{PLL-g-PEG-ssDNA}}}{\sigma^{\mathrm{CBiU}}_{\mathrm{PLL-g-PEG-ssDNA}}}$) and used to evaluate the molecular distribution. Similar to previous observations, the observed distribution scores shift towards 0. Comparing the simulation

(with experimental sampling ratio) with the experimental result, the experimentally observed distribution scores are more positive than the simulated point patterns with the highest degree of dispersity, indicating that the underlying molecular distribution is indeed dispersed despite the undersampling of molecules. This shows that the Clark-Evans test is adequate for the quantification of molecular distribution given the experimental sampling ratio.

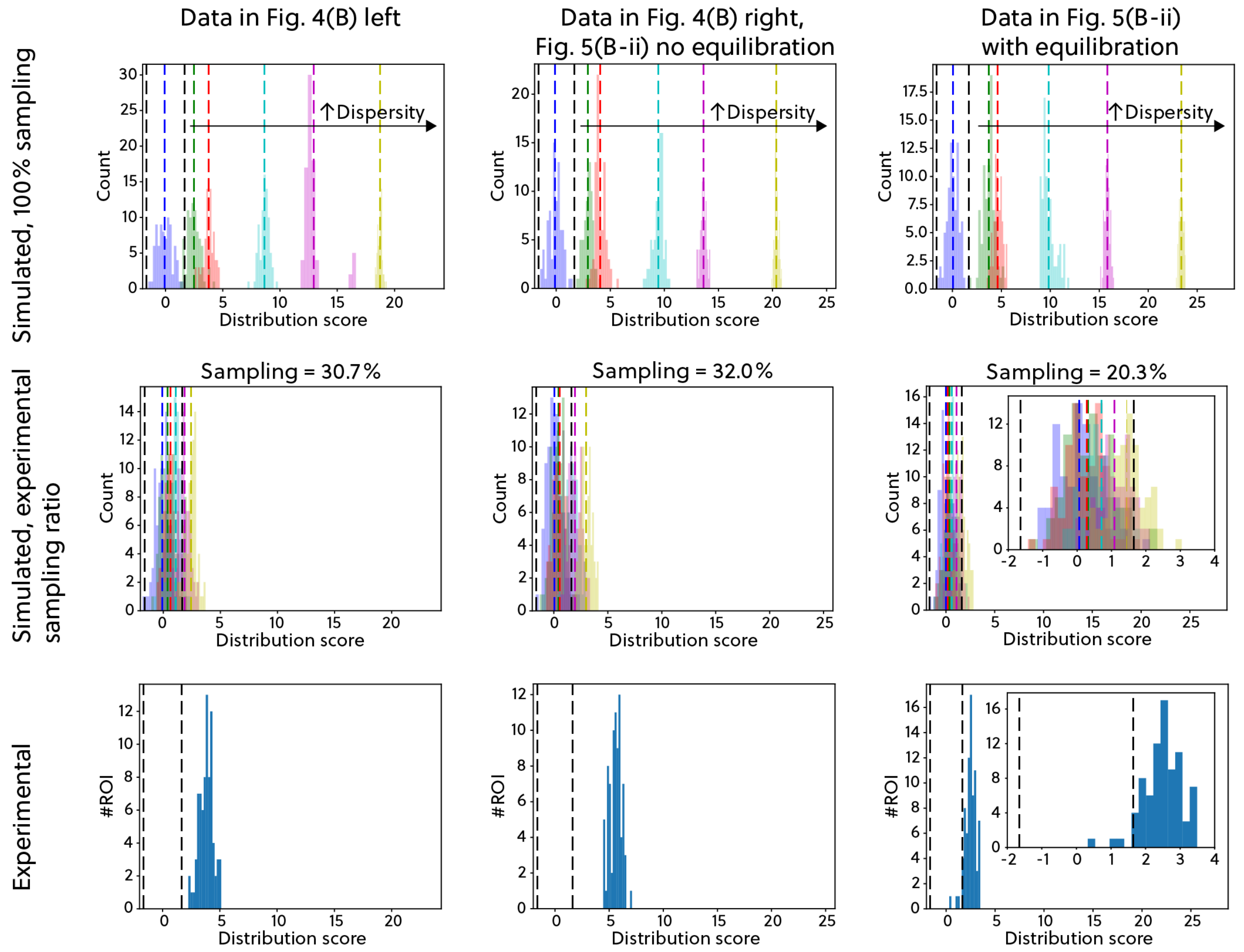

Figure S8: Point patterns were simulated based on the experimentally quantified PLL-g-PEG-ssDNA density $\sigma_{\mathrm{PLL-g-PEG-ssDNA,\ CBiU}}$ and varying degrees of dispersion. Histograms of the observed distribution scores from the simulated point pattern with full sampling of molecules (top row) and experimental sampling ratio (middle row) are plotted. The experimentally quantified distribution scores (bottom row) show a higher distribution score than the simulated point patterns with the highest degree of dispersion. The insets shown are the zoomed-in plot of the histograms.

4 Ionic strength dependence of the spatial molecular heterogeneity

Studies have shown that the SPAAC click chemistry is dependent on the azide or alkyne structures, and on the nature of the buffer used (organic or aqueous), but not on the ionic strength of the buffer.[S15] However, upon performing DNA-PAINT imaging and the analysis framework outlined in this work on biofunctionalized surfaces prepared from buffers of varied ionic strength, we found a pronounced difference in the $\sigma^{\mathrm{DC}}_{\mathrm{PLL-g-PEG-ssDNA}}$ and $\sigma^{\mathrm{CBiU}}_{\mathrm{PLL-g-PEG-ssDNA}}$ density values for the two surfaces.

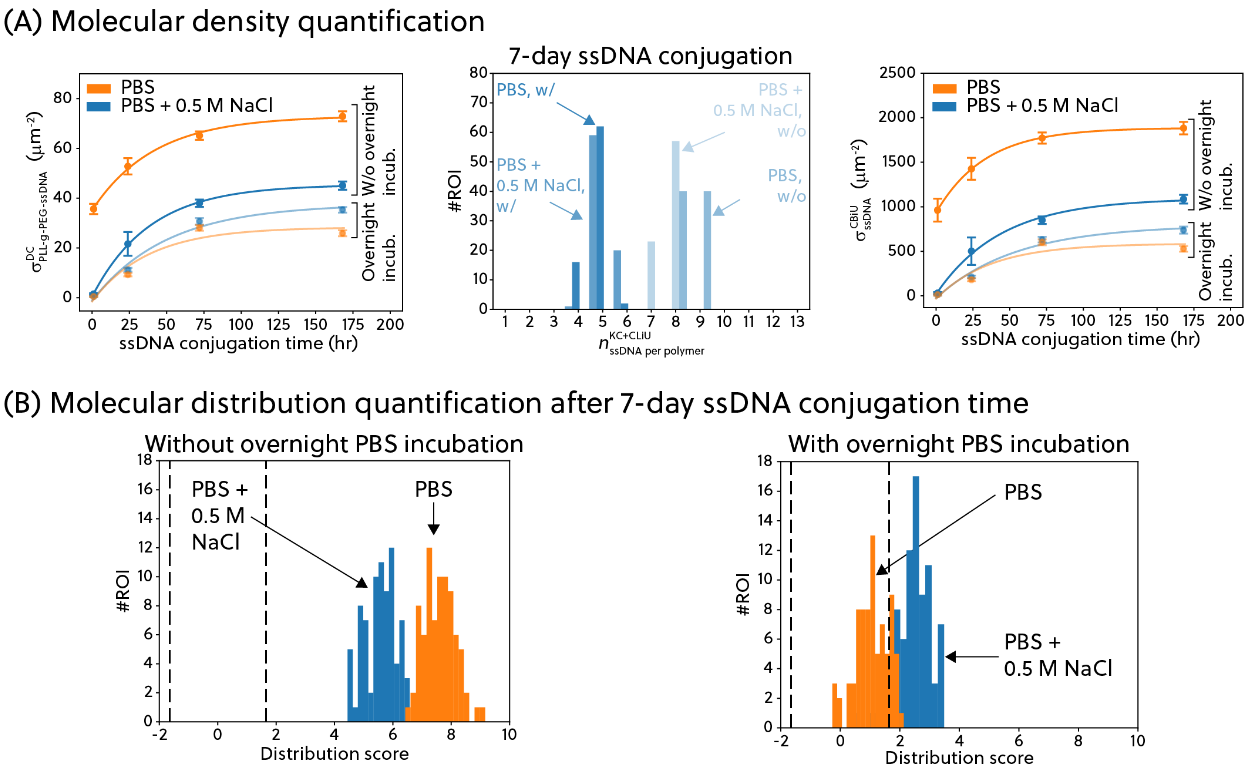

Figure S9: (A) Molecular density of PLL-g-PEG-ssDNA and ssDNA binders and average ssDNA binder per PLL-g-PEG molecule were analyzed for biofunctionalized surfaces prepared with varying ionic strength in the conjugation buffer and/or with the addition of an overnight PBS incubation step. (B) Histograms of the quantified distribution scores for biofunctionalized surfaces with varying preparation conditions after 7 days of conjugation time.

Fig. S9A shows that both quantified densities for the biofunctionalized surface prepared from PBS are significantly higher than those for the surface prepared from PBS supplemented

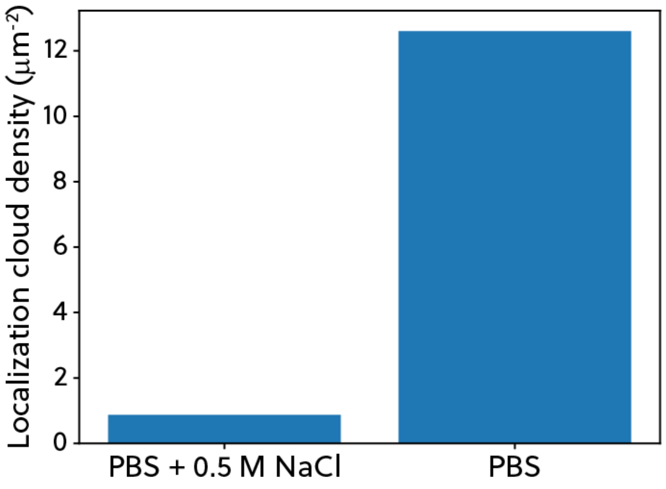

Figure S10: Control samples were prepared by click-coupling the non-complementary docking strands to the polymer layer on the surface in two varying salt conditions for 1 day. The density of the localization cloud found for the sample prepared in PBS supplemented with 0.5 M NaCl is significantly lower than that found for the sample prepared in PBS, indicating more non-specific interactions observed for a low salt condition.

with 0.5 M NaCl at all conjugation times. Although not as drastic, $n^{\mathrm{KC+CLiU}}_{\mathrm{ssDNA\ per\ polymer}}$ for the surface prepared from PBS is also higher than that of the surface prepared from PBS supplemented with extra salt. These unexpected observations raise a question about whether the additional salt in the reaction solution is affecting another molecular component in the system, i.e., the PLL-g-PEG layer. Indeed, we found that the control surface prepared from only PBS shows a 12-fold increase in DC localization cloud density than that prepared from PBS supplemented with salt, as seen in Fig. S10. Furthermore, the difference in densities arising from salt conditions is much reduced if the PLL-g-PEG-coated surface is incubated overnight in PBS prior to ssDNA conjugation.

These observations can be explained by considering the three molecular mechanisms occurring after the PLL-g-PEG adsorption as proposed in this work. Without performing the additional overnight PBS incubation step, all three molecular mechanisms could occur simultaneously. In this regard, the PLL-g-PEG rearrangement and the electrostatic interaction between ssDNA binders and exposed lysine moieties on the PLL-g-PEG molecule

would be impacted by the ionic strength of the buffer solution. Considering the case where a lower ionic strength solution is used, PLL-g-PEG molecules may rearrange more quickly, but ssDNA binder molecules may also experience a stronger electrostatic attraction toward the exposed lysine groups, thus hindering the PLL-g-PEG molecules from forming a well-structured, low-fouling surface. Since we observe a larger number of non-specific interactions for the control surface with low ionic strength, we speculate that the rate at which the ssDNA binders are non-specifically interacting with the PLL-g-PEG molecules is higher than that at which the PLL-g-PEG molecules rearrange. By allowing the PLL-g-PEG molecule to rearrange during the overnight PBS incubation step, the number of exposed lysine moieties on the PLL-g-PEG molecules may be reduced, hence reducing the ionic strength dependence of the increase of PLL-g-PEG-ssDNA density and ssDNA binder density over conjugation time.

Meanwhile, we do not observe a strong ionic strength dependence on the molecular distribution of the PLL-g-PEG-ssDNA molecules, see Fig. S9B. In general, the surfaces prepared with the overnight PBS incubation step provide a more homogeneous biofunctionalized surface with a lower distribution score.

5 Dispersion of PLL-g-PEG-ssDNA molecules

In this study, we observed that the PLL-g-PEG-ssDNA molecules are more dispersed when the ssDNA conjugation duration increases, see Fig. S11A. It is not a priori clear what could cause this observation, but we hypothesized that the increase in ssDNA binders per PLL-g-PEG molecule over time creates a steric repulsive force between neighboring polymer molecules, resulting in a more dispersed molecular distribution over time. However, on closer inspection of the experimental data, we found that the increase in dispersity of the molecules only occurred for biofunctionalized surfaces that were prepared without the overnight PBS incubation step. Should the increase in observed molecular dispersity be caused by the steric repulsive force of the PLL-g-PEG-ssDNA molecules, we should also observe a similar trend for the surfaces prepared with the additional overnight PBS incubation step considering that the quantified PLL-g-PEG-ssDNA densities are similar for both surfaces. This hints that the steric repulsive force may not be the sole contributor to this observation.

Attempting to understand this phenomenon, we explored another hypothesis: the observed increase in dispersity may be caused by the inherent molecular distribution of the azide-functionalized PLL-g-PEG molecules. Assuming that the azide-functionalized PLL-g-PEG molecules are inherently dispersed before the conjugation of ssDNA binders, more of these molecules were being imaged in DNA-PAINT as more ssDNA binders were conjugated to them over time. To explore this hypothesis, we performed Monte Carlo simulations to study this phenomenon. Firstly, point patterns of azide-functionalized PLL-g-PEG molecules were simulated for varying molecular distributions. These generated spatial locations were then sampled randomly to simulate the conjugation of ssDNA binders to the polymer molecules over time. These sub-sampled points are the molecules that can be imaged during a typical DNA-PAINT measurement. Moreover, the undersampling of molecules due to experimental limitations was also taken into account during the simulation.

Fig. S11B shows that the observed distribution scores tend towards the simulated underlying molecular distribution of the azide-functionalized PLL-g-PEG molecules as ssDNA

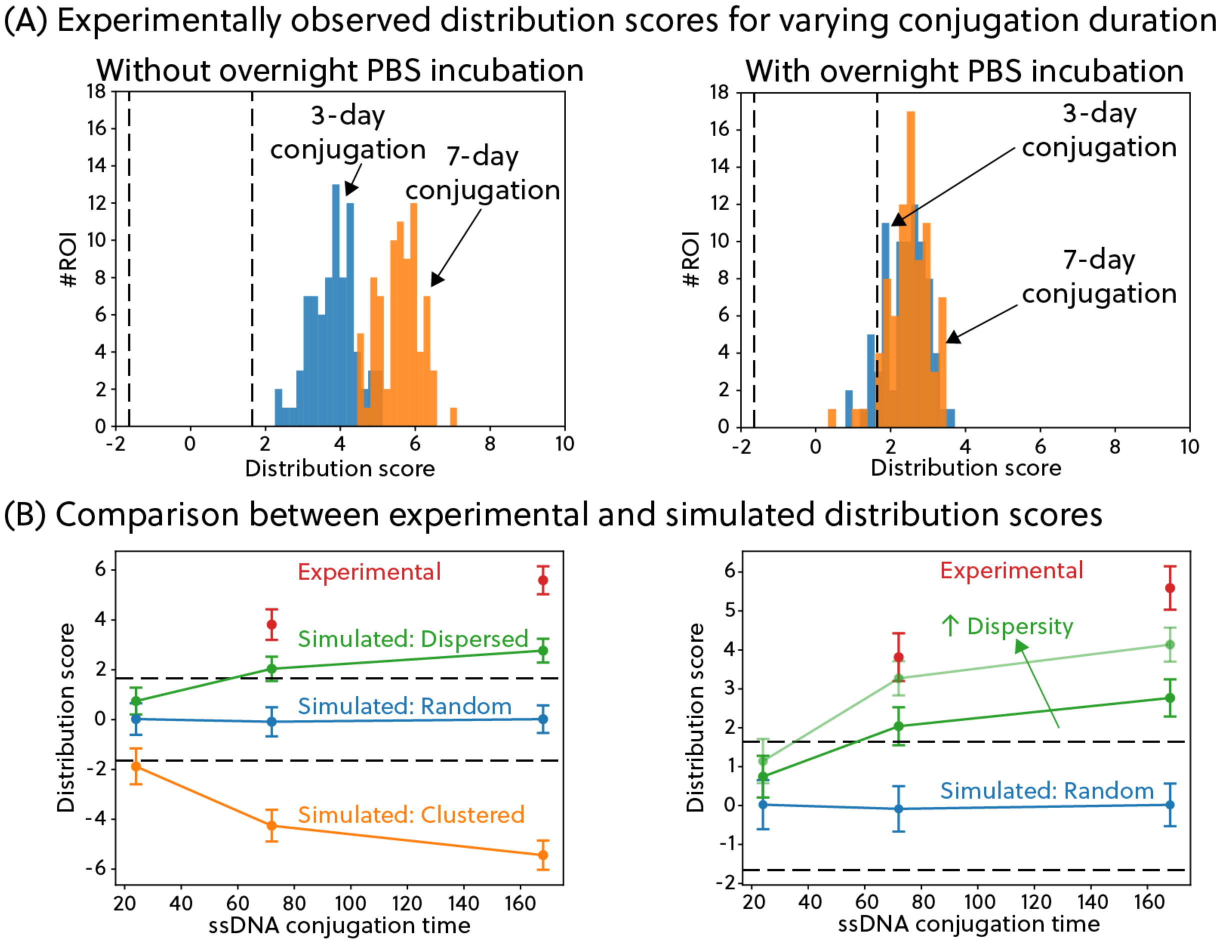

Figure S11: (A) Experimentally observed distribution scores for biofunctionalized surfaces prepared with varying conjugation duration, and with or without the addition of an overnight PBS incubation step. The increase in molecular dispersion is only observed for the surfaces prepared without the additional incubation step. (B) Monte Carlo simulation results are compared with the experimentally observed distribution scores obtained from biofunctionalized surfaces prepared without the additional overnight PBS incubation step. This suggests that the observed increase in molecular dispersion may be caused by the inherent molecular distribution of the azide-functionalized PLL-g-PEG molecules.

conjugation time increases. As the degree of dispersity of the inherent molecular distribution increases, the observed distribution scores are also more positive over time. This suggests that it is plausible that the experimentally observed increase in dispersion is caused by the underlying molecular distribution of the azide-functionalized PLL-g-PEG molecules. Since the trend is not observed for surfaces prepared with the additional overnight PBS incubation step, we hypothesize that this extra biofunctionalization step allowed the azide-

functionalized PLL-g-PEG molecules to not only adopt an equilibrium conformation that is low-fouling but also an equilibrium distribution that is only slightly dispersed. On the other hand, when the PLL-g-PEG molecules are not allowed to equilibrate, we postulate that the non-specific conjugation of the ssDNA binder molecules towards the unbound lysine on the polymer molecules may "lock" the individual polymer molecules in place, resulting in a more dispersed distribution. However, it remains unclear as to why the azide-functionalized PLL-g-PEG molecules would adopt a dispersed distribution during the adsorption process.

Overall, the observed molecular distribution seems to be a result of the complex interplay between the intermolecular forces in the model system used in this study. With the technique and analysis outlined in this work, we cannot conclude whether the observed increase in dispersity is caused by the steric repulsion between the ssDNA-functionalized PLL-g-PEG molecules, or by the inherent molecular distribution of the azide-functionalized PLL-g-PEG molecules, or by a combination of both. Since this work aims to demonstrate a methodology that is capable of quantifying molecular distribution, it is not within the scope of this work to fully investigate and uncover the complex intermolecular dynamics at play in the model system.

6 Additional information and extended data

6.1 ssDNA sequences

Table S6 shows the ssDNA molecules that were used in this work. Sequence #1 is pre-hybridized with Sequence #2 or Sequence #3 to prepare the binder-functionalized surface and the control surface respectively. Sequence #4 is the ssDNA imager strand.

Table S6: ssDNA sequences used in this work.

#	5' end group	Sequence	3' end group
1	None	CGATTCGAGAACGTGACTGCTTTTT	DBCO
2	None	GCAGTCACGTTCTCGAATCGAACATTATTACA	None
3	None	GCAGTCACGTTCTCGAATCGAAGTAATAATG	None
4	ATTO647N	TTGTAATAATG	None

6.2 Investigation of non-specific interactions for control surfaces

Three surfaces coated only with PLL-g-PEG molecules were imaged using DNA-PAINT to study the non-specific interactions between the imager strands and the control surfaces. Fig. S12 shows the time-aggregated localization plot for the control surfaces. We observed a large number of non-specific interactions when the PLL-g-PEG-containing solution was allowed to incubate on the glass surfaces for 3 hours or overnight. However, a drastic reduction in non-specific interactions was observed when the PLL-g-PEG-containing solution was incubated for 3 hours, followed by an exchange of solution and an overnight incubation in PBS. This suggests that the non-specific interactions are not caused by insufficient coating of the PLL-g-PEG molecules but by the disruption of the equilibrium conformation of the coated PLL-g-PEG polymer layer from the exchange of solution. By performing an additional incubation step, the attached PLL-g-PEG molecules can dynamically rearrange to form an equilibrium conformation that is low-fouling.

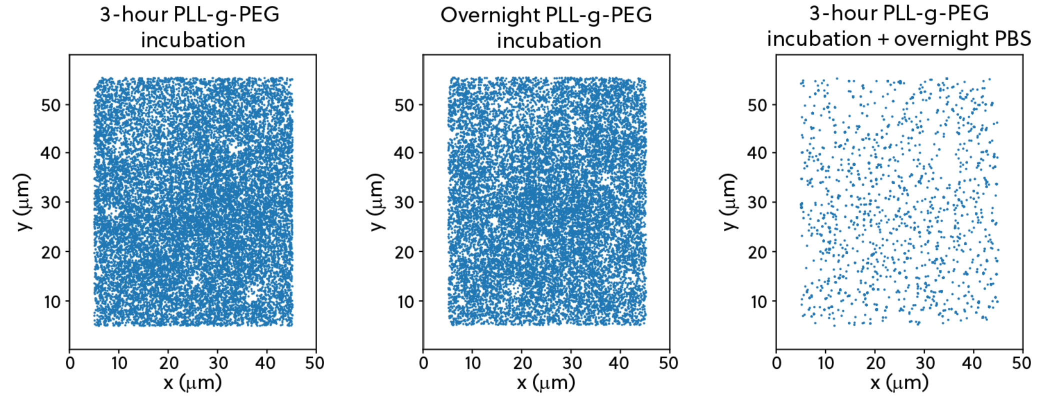

Figure S12: A large number of non-specific interactions is observed for the surfaces with a 3-hour (left) or overnight (middle) PLL-g-PEG incubation step. When the an additional overnight PBS incubation step is performed, the control surface (right) showed much reduced non-specific interactions.

On top of that, control surfaces prepared from non-complementary ssDNA molecules are used to further validate the occurrence of the SPAAC conjugation process. The control surfaces were prepared via coating the cleaned glass surface with 1% v/v PLL-g-PEG-N3/PLL-g-PEG solution for 3 hours, followed by the incubation of the DBCO-functionalized

non-complementary ssDNA molecules for varying duration. The surfaces were analyzed using the direct counting approach to obtain the density of localization cloud/non-specific interaction sites. If the ssDNA molecules were not conjugated on the PLL-g-PEG-coated surface, we should observe no difference between the positive control surfaces (samples with 10bp complementary ssDNA molecules) and the negative control surfaces (samples with non-complementary ssDNA molecules). As shown in Fig. S13(A), a clear difference between the negative control surfaces and the positive control surfaces can be observed for all conjugation duration (except for the samples prepared from 1-hour conjugation duration). Zooming in on the negative control surfaces (Fig. S13(B)), the density of non-specific interaction sites decreases as conjugation duration increases. We attribute this observation to the increase in ssDNA molecules conjugated on the surface, resulting in a larger electrostatic repulsion between the ssDNA molecules and the imager strands.

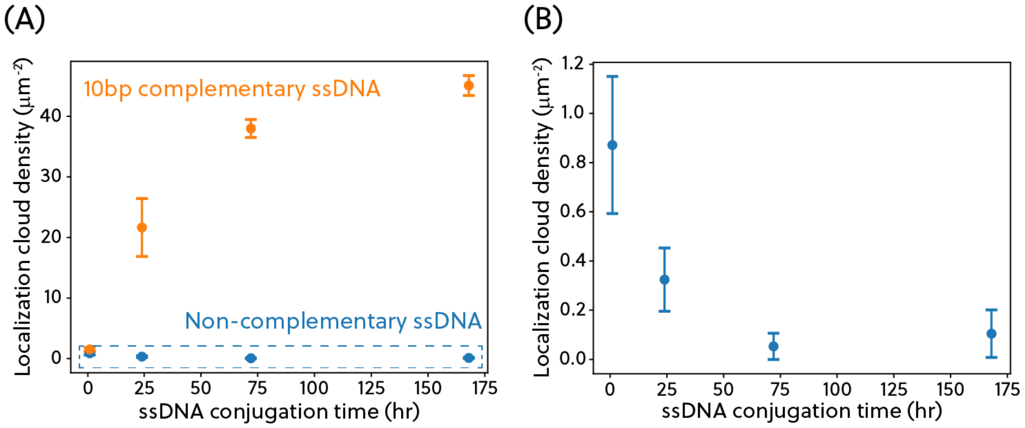

Figure S13: (A) Localization cloud density increases with ssDNA conjugation duration for the positive control surfaces (samples prepared with 10bp complementary ssDNA molecules). Clear difference between the positive control surfaces and the negative control surfaces (samples prepared with non-complementary ssDNA molecules) for conjugation time $\geq$ 1 day can be observed. (B) Only the data for the negative control surfaces are shown. For the negative control surfaces, the localization cloud density decreases with ssDNA conjugation duration.

6.3 Application of Resolution Enhancement by Sequential Imaging for densely-functionalized sample

Resolution Enhancement by Sequential Imaging (RESI) is a DNA barcoding method based on DNA-PAINT to improve the resolution of super-resolution fluorescence microscopy down to the Ångstrom scale.[S16] To image the molecules that are spaced closer than the DNA-PAINT resolution, RESI adopts a sequential imaging approach to resolve these molecules by stochastically labeling these molecules with n orthogonal docking strands, implying that n imaging rounds are required to fully resolve the molecules of interest. There are two cases to consider in order to apply RESI on high-density samples:

1. The molecules of interest are spaced more closely than the standard DNA-PAINT resolution, but dense regions of these molecules are located sparsely on a larger area. Examples of such molecules include protein oligomers, ssDNA binder molecules functionalized densely on a relatively short chain polymer PLL-g-PEG (as is in this article), *etc.*

2. The molecules of interest are distributed randomly (in a CSR fashion) at a certain density. At high density, the intermolecular distance becomes less than the standard DNA-PAINT resolution, hence the molecules can no longer be resolved in DNA-PAINT imaging.

We note that the molecular system studied in this article is a combination of the two cases. The ssDNA binder molecules are conjugated densely on a single PLL-g-PEG molecule (the estimated size of the PLL-g-PEG molecule is near the DNA-PAINT resolution limit) and the ssDNA-functionalized PLL-g-PEG molecules are functionalized at a high density (in the range of $10^2\ \mu m^{-2}$ to $10^3\ \mu m^{-2}$) on the glass substrate.

In the first case, to resolve a set of an arbitrary number of molecules m spaced more closely than the DNA-PAINT resolution, the proportion of resolvable sets of molecules follows the

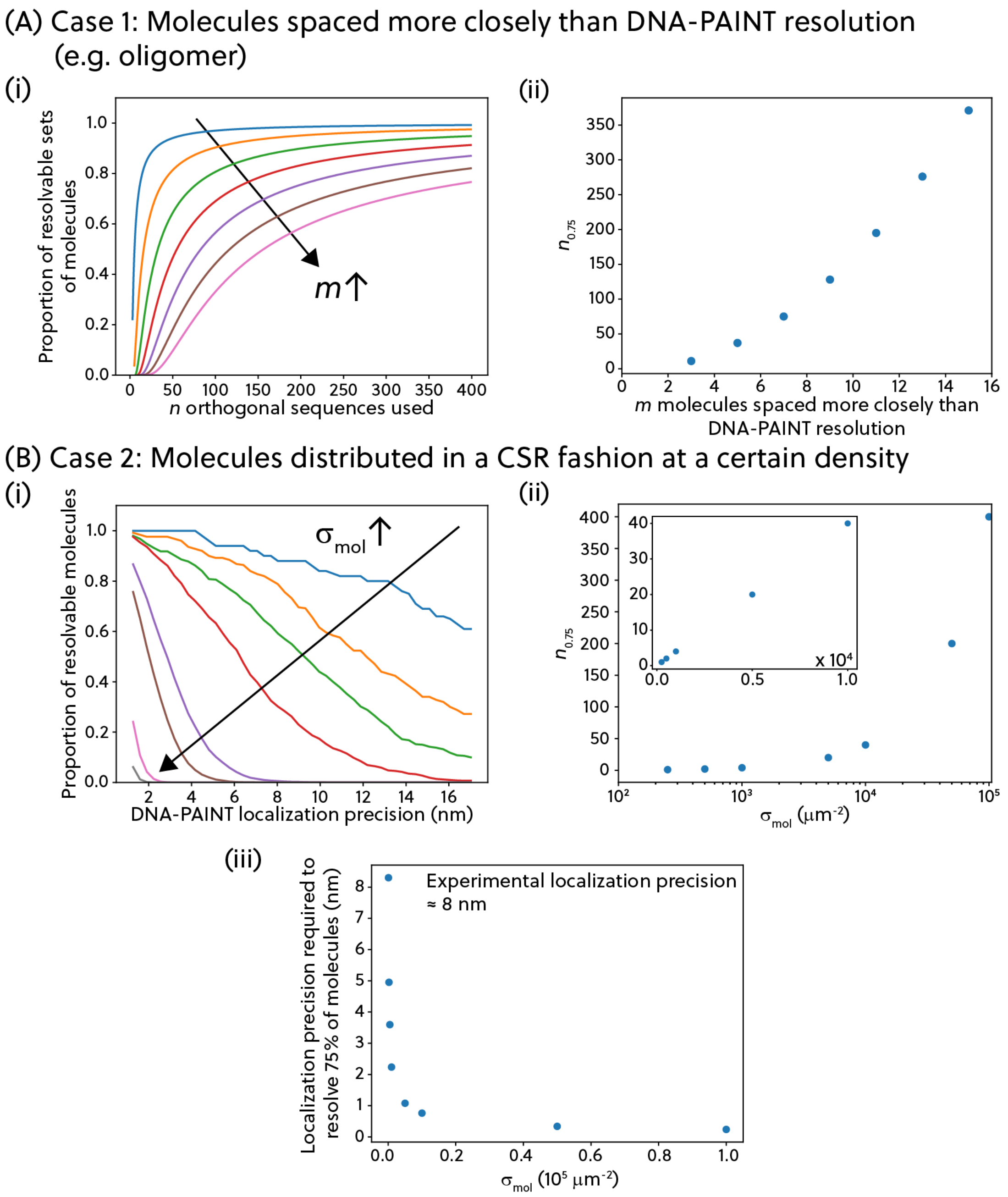

Figure S14: (A) Case 1: the molecules are spaced more closely than the DNA-PAINT resolution. The proportion of resolvable sets of molecules plotted against n for varying m (i) while the number of n orthogonal sequences required to achieve 75% resolvable sets of molecules is plotted against m (ii). (B) Case 2: The molecules are distributed randomly at a certain density. The proportion of resolvable molecules at a given DNA-PAINT localization precision drastically decreases with increasing molecular densities (i). For increasing molecular densities, the number of n imaging rounds/orthogonal sequences required to resolve 75% of the molecules (ii) increases, while the localization precision required to resolve 75% of the molecules (iii) decreases.

equation,

$$\mathrm{P}(m,\ n) = \frac{n!}{(n-m)!n^m}. \tag{S21}$$

Exploring this relation, Fig. S14A shows that P reduces as the number of molecules m in the resolution-limited area increases. Consider a densely functionalized surface ($m > 5$), more than 50 orthogonal docking strands are required to resolve 75% of the closely spaced molecules, highlighting the requirement to have more than 50 imaging rounds to study high-density samples.

To explore the use case of RESI in the second scenario, random distributions of molecules were simulated based on varying molecular density σ_{mol}, and the nearest neighbor distances of the molecules were calculated. For a given density, nearest neighbor distances that are above a certain resolution (2.35 × DNA-PAINT localization precision) were considered as the molecules that can be resolved by the given DNA-PAINT localization precision, giving the proportion of resolvable molecules for a given molecular density and DNA-PAINT localization precision. Fig. S14B shows that the proportion of resolvable molecules reduces drastically as the molecular density σ_{mol} increases. RESI works by splitting the molecules of interest into different stochastically labeled subsets, hence reducing the effective molecular density *via*

$$\sigma_{\mathrm{mol,eff}} = \frac{\sigma_{\mathrm{mol}}}{n}. \tag{S22}$$

Given the average experimental DNA-PAINT localization precision ($\approx 8\,\mathrm{nm}$ in Fig. S2), we found that the effective molecular density $\sigma_{\mathrm{mol,eff}}$ of which 75% of the molecules can be resolved with the experimental localization precision to be approximately $250\,\mathrm{\mu m^{-2}}$. This value is then used to evaluate the number of orthogonal docking strands/imaging rounds n that are required for resolving at least 75% of molecules for a given molecular density, as shown in Fig. S14B(ii). We observe that the number of imaging rounds increases drastically with the molecular density.

To estimate the acquisition time for one imaging round t_{acq}, we consider the following

equation:[S16]

$$t_{\text{acq}} = \frac{t_{\text{exposure}} \times \sigma^2_{\text{DNA-PAINT}}}{\tau_{\text{b}} \times k_{\text{on}} \times c_{\text{img}} \times \sigma^2_{\text{RESI}}} \tag{S23}$$

where t_{exposure} denotes exposure time, $\sigma_{\text{DNA-PAINT}}$ DNA-PAINT localization precision, τ_{b} characteristic bound state lifetime, k_{on} DNA molecular association rate, c_{img} imager concentration, and σ_{RESI} RESI localization precision. The RESI localization precision is taken as the localization precision required to resolve at least 75% of the molecules, as shown in Fig. S14B(iii). Given the experimental parameters used in this article (see Table S7), the acquisition time for one imaging round is estimated to range from 1 hour up to 50 days in order to image at least 75% of the molecules for molecular densities ranging from $10^2\,\mu\text{m}^{-2}$ to $10^5\,\mu\text{m}^{-2}$. Since the total acquisition time scales with the number of imaging rounds ($t_{\text{acq}} \times n$), we expect a long acquisition time to implement RESI for high-density samples. Furthermore, since the localization precision required to resolve 75% of molecules is inversely proportional to the square of the molecular density (Fig. S14B(iii)), it is good to note that the acquisition time for one imaging round thus scales with the square of the molecular density.

Table S7: Parameters used to estimate the acquisition time required for a given molecular density.

t_{exposure}	(s)	0.1
τ_{b}	(s)	1
k_{on}	($\text{M}^{-1}\text{s}^{-1}$)	10^6
c_{img}	(M)	2.5×10^{-11}
$\sigma_{\text{DNA-PAINT}}$	(nm)	8

There are a few strategies to speed up DNA-PAINT imaging such as performing simultaneous multicolor imaging,[S17] optimizing buffer conditions,[S18] employing period DNA sequence motifs as the docking strands,[S19] *etc.* Despite these potential speed improvements, we still expect the total acquisition time to be in the order of hours (up to a few days) for imaging densely-functionalized samples (for molecular density up to $10^5\,\mu\text{m}^{-2}$). Therefore, due to the long acquisition time required, we deemed it unrealistic to implement RESI for

this study.

References

(S1) Kenausis, G. L.; Vörös, J.; Elbert, D. L.; Huang, N.; Hofer, R.; Ruiz-Taylor, L.; Textor, M.; Hubbell, J. A.; Spencer, N. D. Poly(l-lysine)-g-Poly(ethylene glycol) Layers on Metal Oxide Surfaces: Attachment Mechanism and Effects of Polymer Architecture on Resistance to Protein Adsorption. *Journal of Physical Chemistry B* **2000**, *104*, 3298–3309.

(S2) Heuberger, M.; Drobek, T.; Spencer, N. D. Interaction Forces and Morphology of a Protein-Resistant Poly(ethylene glycol) Layer. *Biophysical Journal* **2005**, *88*, 495–504.

(S3) Feuz, L.; Leermakers, F. A. M.; Textor, M.; Borisov, O. Adsorption of Molecular Brushes with Polyelectrolyte Backbones onto Oppositely Charged Surfaces: A Self-Consistent Field Theory. *Langmuir* **2008**, *24*, 7232–7244.

(S4) Mou, J.; Czajkowsky, D. M.; Zhang, Y.; Shao, Z. High-resolution Atomic-force Microscopy of DNA: the Pitch of the Double Helix. *FEBS Letters* **1995**, *371*, 279–282.

(S5) Riera, R.; Archontakis, E.; Cremers, G. A.; De Greef, T. F. A.; Zijlstra, P.; Albertazzi, L. Precision and Accuracy of Receptor Quantification on Synthetic and Biological Surfaces using DNA-PAINT. *ACS Sensors* **2023**, *8*, 80–93.

(S6) Fukunaga, K.; Hostetler, L. The Estimation of the Gradient of a Density Function, with Applications in Pattern Recognition. *IEEE Transactions on Information Theory* **1975**, *21*, 32–40.

(S7) Cheng, Y. Mean Shift, Mode Seeking, and Clustering. *IEEE Transactions on Pattern Analysis and Machine Intelligence* **1995**, *17*, 790–799.

(S8) Comaniciu, D.; Meer, P. Mean Shift: A Robust Approach Toward Feature Space Analysis. *IEEE Transactions on Pattern Analysis and Machine Intelligence* **2002**, *24*, 603–619.

(S9) Pedregosa, F.; Varoquaux, G.; Gramfort, A.; Michel, V.; Thirion, B.; Grisel, O.; Blondel, M.; Prettenhofer, P.; Weiss, R.; Dubourg, V.; Vanderplas, J.; Passos, A.; Cournapeau, D.; Brucher, M.; Perrot, M.; Duchesnay, SciKit-Learn: Machine Learning in Python. *HAL (Le Centre pour la Communication Scientifique Directe)* **2011**,

(S10) Jungmann, R.; Avendaño, M. S.; Dai, M.; Woehrstein, J. B.; Agasti, S. S.; Feiger, Z.; Rodal, A. A.; Yin, P. Quantitative Super-resolution Imaging with qPAINT. *Nature Methods* **2016**, *13*, 439–442.

(S11) Stein, J. C.; Stehr, F.; Schueler, P.; Blumhardt, P.; Schueder, F.; Mücksch, J.; Jungmann, R.; Schwille, P. Toward Absolute Molecular Numbers in DNA-PAINT. *Nano Letters* **2019**, *19*, 8182–8190.

(S12) Dey, S.; Rivas-Barbosa, R.; Sciortino, F.; Zaccarelli, E.; Zijlstra, P. Biomolecular Interactions on Densely Coated Nanoparticles: A Single-molecule Perspective. *Nanoscale* **2024**,

(S13) Clark, P. J.; Evans, F. C. Distance to Nearest Neighbor as a Measure of Spatial Relationships in Populations. *Ecology* **1954**, *35*, 445–453.

(S14) Fröhlich, M.; Quednau, H. D. Statistical Analysis of the Distribution Pattern of Natural Regeneration in Forests. *Forest Ecology and Management* **1995**, *73*, 45–57.

(S15) Dommerholt, J.; Rutjes, F. P. J. T.; Van Delft, F. L. Strain-Promoted 1,3-Dipolar Cycloaddition of Cycloalkynes and Organic Azides. *Topics in Current Chemistry* **2016**, *374*.

(S16) Reinhardt, S.; Masullo, L. A.; Baudrexel, I.; Steen, P.; Kowalewski, R.; Eklund, A. S.; Strauss, S.; Unterauer, E. M.; Schlichthaerle, T.; Strauss, M. T.; Klein, C.; Jungmann, R. Ångström-resolution Fluorescence Microscopy. *Nature* **2023**, *617*, 711–716.

(S17) Gimber, N.; Strauss, S.; Jungmann, R.; Schmoranzer, J. Simultaneous Multicolor DNA-PAINT without Sequential Fluid Exchange Using Spectral Demixing. *Nano Letters* **2022**, *22*, 2682–2690.

(S18) Schueder, F.; Stein, J.; Stehr, F.; Auer, A.; Sperl, B.; Strauss, M. T.; Schwille, P.; Jungmann, R. An Order of Magnitude Faster DNA-PAINT Imaging by Optimized Sequence Design and Buffer Conditions. *Nature Methods* **2019**, *16*, 1101–1104.

(S19) Strauss, S.; Jungmann, R. Up to 100-fold Speed-up and Multiplexing in Optimized DNA-PAINT. *Nature Methods* **2020**, *17*, 789–791.
